# OpenWater Hub: An open-source hardware platform for marine robotics support and water quality data acquisition

**DOI:** 10.1016/j.ohx.2026.e00845

**Published:** 2026-10-01

**Authors:** Hany A. Elesawy, Ahmed I. Ahmed, Yahia A. AboZaid, Lobna A. Said, Irene Samy, Ahmed G. Radwan

**Affiliations:** a Bio-Hybrid Soft Robotics Laboratory (BHSRL), Nile University, Sheikh Zayed City, 12588, Egypt; b Smart Engineering Systems Research Center (SESC), Nile University, Sheikh Zayed City, 12588, Egypt; c Nanoelectronics Integrated Systems Center (NISC), Nile University, Sheikh Zayed City, 12588, Egypt; d School of Engineering and Applied Sciences, Nile University, Giza, 12588, Egypt; e Department of Mechanical Engineering, Al-Azhar University, Cairo, 11651, Egypt; f Department of Engineering Mathematics and Physics, Cairo University, Giza, 12613, Egypt

**Keywords:** Open-source hardware, Marine robotics, Soft robotics, Water quality monitoring, Internet of Things (IoT), Edge computing

## Abstract

At present, there is no single open-source electronic hardware platform that natively integrates 24 V industrial RS485 sensors with marine robotic and hydroponic actuation. Consequently, hardware fragmentation exists severely in underwater sensing and environmental monitoring. Researchers using benchtop calibration must purchase closed-source ecosystems with proprietary operating systems, limiting the use of custom control loops. Conversely, open-source embedded systems provide optimized low-voltage passive monitoring but lack sufficient power for heavy actuators. The OpenWater Hub (OWH) provides a centralized platform for these two areas to exist on one printed circuit board (PCB). The PCB features a 24 V pass-through bus that supports the heavy inrush currents of industrial RS-485 probes. A dual-ATmega128A topology is utilized to prevent serial polling delays from disrupting real-time motor control. The architecture consists of one chip that isolates Modbus communication for sub-millisecond parsing, and another chip that drives soft robotic fish and actuators via four 3.0 A PWM channels. The core board is configured to transform across four deployment variants: a benchtop logger, a portable field laboratory, an autonomous floating station, and a tethered robotic support hub. Modular UART sockets support telemetry via LoRa, Wi-Fi, Bluetooth, and cellular modules. The static baseline power consumption was 196.4 mW; after 240 h of thermal testing, the OpenWater Hub demonstrated >99.97% telemetry reliability and logged chemical validations yielding a 0.9903 correlation coefficient value. Documentation and C++ logic files for the reproduction of all deployment variants are included.

## Highlights

• Integrates 24 V RS485 Modbus sensing with 3.0 A PWM marine robotic actuation.
• Dual-MCU topology isolates serial polling latency from real-time motor control.
• Single printed circuit board scales across four distinct deployment variants.
• Hardware sustained >99.97% telemetry delivery over a 240-hour thermal test.
• Includes reproducible electronic schematics, mechanical CAD, and C++ firmware.

## Graphical abstract

**Figure:**
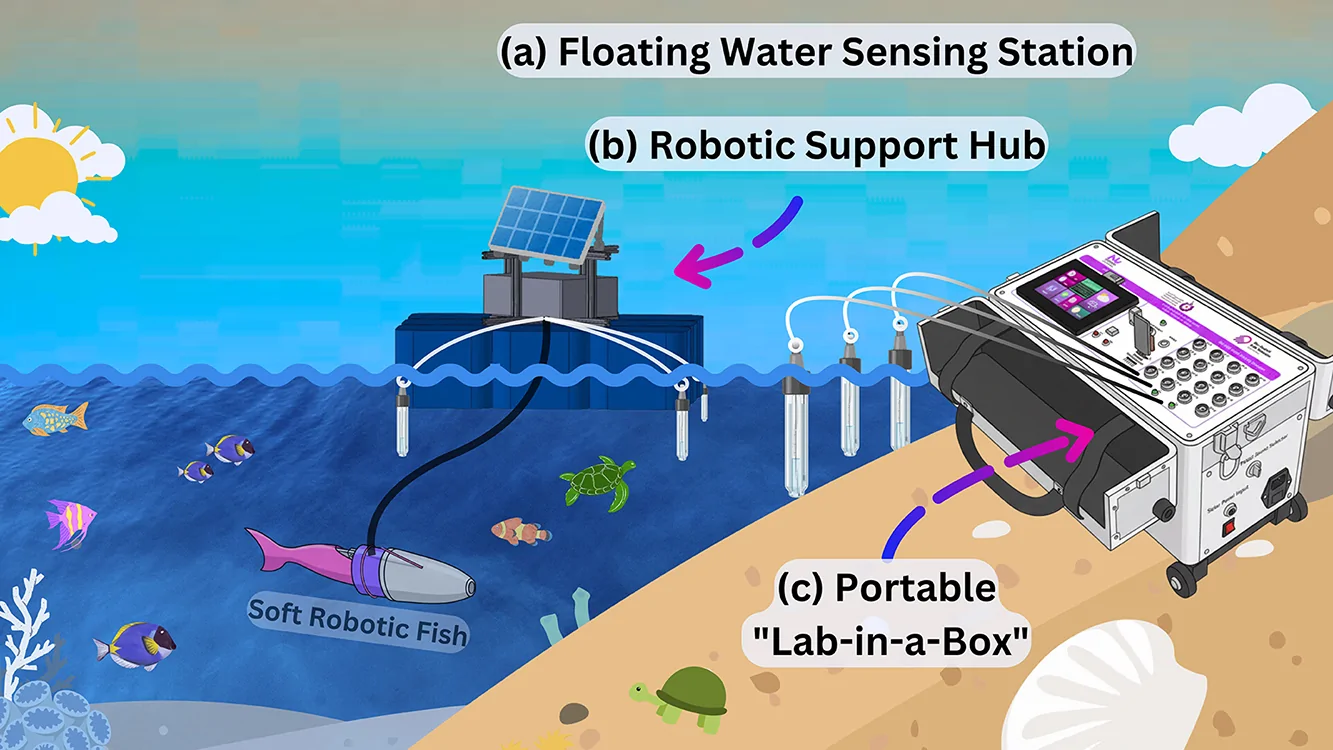

**Table:** 

**Specifications table**	

Hardware name	OpenWater Hub

Subject area	Engineering and material science

Hardware type	Marine Robotics and Sensing

Closest commercial analog	No single commercial analog available; replaces separate water quality meters, data loggers, and robotic command relays.

Open source license	CC-BY 4.0

Cost of hardware	$34.75 (Data Node PCB); $45.60 (Unified PCB); $292.60 (Portable Lab); $475.60 (Floating Station)

Source file repository	https://doi.org/10.17605/OSF.IO/KMNYA https://github.com/h3esawy/OpenWaterHub

## 1 Hardware in context

The total contribution of the ocean to the global gross domestic product is about 2.5% [1], and a large portion of that comes from coral reefs. Coral reefs are home to at least one-quarter of all marine species [2]; therefore, they also provide a large amount of money for local communities each year [3]; and as such, in order to achieve the goals of the United Nations Sustainable Development Goal 14 (Life Below Water) and the Global Ocean Observing System (GOOS), we need continuous, very dense, spatial data. There are limitations to the traditional laboratory-based methods used for analyzing water quality. Laboratory-based methods introduce delays by providing temporal information [4], while remote sensing can image the entire surface of bodies of water but cannot penetrate into the body of the water [5].

The literature has shown that there are various forms of mobile platforms for obtaining physical in-situ data; for example, aerial systems can obtain targeted surface samples [6]. Surface spatio-temporal data can be mapped with either robotic airboats [7], or autonomous boats [8]. Subsurface data can be obtained from either biomimetic robotic dolphins [9], or soft robotic fish which utilize specific path-planning techniques [10]. Soft robots allow researchers to have physical contact with the coral reef structure while not disturbing the marine environment around it [11]. Models of open-fabrication enable wider academic accessibility to this particular form of locomotion [12], [13].

Marine vehicles require standardized electronic interfaces to operate varied sensor bundles [14]. One application is the RS485 Modbus protocol, which is preferred in industrial applications due to its reliable performance in harsh environments and over distances that reach 1200 m [15], so it is typically relied on for hardware-level sensor communication. Translating this local serial data to cloud-based MQTT brokers is a standard structural method [16], [17], [18], [19]. These RS485 networks support low-power, distributed sensing units [20] across disciplines [21]. Integrating these communication standards enables legacy ships and research vehicles to adopt open interoperability frameworks [22], [23].

Deployment of the communication gateway offshore requires accurate control of energy management. The low-consumption embedded microcontroller (MCU) maximizes operation time [24] and, as such has to have large-capacity energy storage or another method to activate propulsion continuously, that are usually required by station-keeping buoys [25], [26]. In some cases, it will be necessary to use solar power for surface-based data acquisition systems to recharge controllers and maintain sufficient battery reserves to support long deployments or during severe weather [27], [28].

Commercial platforms meet the needs of marine monitoring’s differential signaling and voltage requirements (as we will discuss), however, they are constrained by very limited capabilities of how users can interact with them. High-end equipment, which includes the YSI EXO3 multi-parameter sonde [29] and Yosemitech probes [30] are capable of transmitting native data using open industrial communication protocols, such as Modbus RTU (RS485) or SDI-12. Vendor lock-in is generally confined to the data logger and infrastructure levels. As seen throughout all marine-related industries, there exists a form of proprietary restrictions. For example, companies like NexSens [31], [32] produce stable surface buoys that can be used for years. Manufacturers of industrial-grade logging equipment include Aqualabo [33], In-Situ [34], Seeed Studio [35], and Digital Matter [36]. Each of these products are based on a proprietary real-time operating system (RTOS), which means that the user has virtually no access to customize anything. Examples of commercially available hydroponic and smart-agricultural ecosystems (i.e., ReNile iRoots [37]) illustrate this point. The ReNile iRoots product uses common, off-the-shelf RS485 Modbus probes to collect data. However, the manufacturer does not release the register map associated with each probe, so a researcher will have to develop their own way to extract the relevant information contained in each probe’s digital payload. When using the closed ecosystem, the user is subjected to significant physical hardware partitioning and artificially restricted access to their data. To gain simple kinematic control over the system, the researcher will have to purchase additional, expensive stand-alone modules, for example, a five-channel relay module that drives the total system expenditure near $1600 USD. Additionally, the closed-cloud architecture limits the researcher’s ability to access their data in high-resolution formats, resulting in having to manually export log files in small segments, each segment containing approximately 1 h of data due to the software constraints. Although the NexSens X-Series loggers provide some advantages through their 12 V switched power supply to turn sensors off when idle to save battery life, the primary function remains as a passive data aggregator. Due to being closed source, it is impossible for a researcher to write custom C++ code to read an optical DO probe while simultaneously commanding an external underwater robot thruster.

Low-cost, open-source systems avoid the commercial cost of development while they focus on low-voltage, floating, passive drifting systems that have been developed, such as camera-mounted systems [38] or float systems [39]. The use of various systems with open-source code has allowed development of multiple applications with many alternative designs. These include OpenMetBuoy [40], ALog [41], GatorByte [42], LOOM [43], HydroMisc [44], microSWIFT [45], VSB [46], GPSB [47] and even specialized drifters such as the XFZ wave buoy [48]. These systems all provide the ability to record analog and I^2^C data from probes at lower cost using I^2^C software sleep commands or 5 V switchable regulators (seen in the ALog) as an example. However, because all these low-cost systems operate off a 3.3 V or 5 V logic rail they do not support the use of digital inputs or outputs such as those used by industrial Modbus 24 V sensors. The same is true of academic RS485 integration devices such as the Jericho logger [49], Nexus platform [50] and Hypnos board [51] since none of them provide their own native 24 V power planes, and therefore require the use of vulnerable external DC-DC converters. Translating RS485 into CAN bus within the OpenDAM architecture [52] incurs an additional loss of 1.5 W of micro-computer power. Finally, hardware employing I^2^C-to-RS485 translators [53] or ESP32-based LoRa RS485 loggers [54], [55], [56] made it possible to achieve protocol communications but ignored physical robotics actuation.

Polling half-duplex RS485 networks demands strict timing determinism, requiring the master node to manually toggle transceiver pins with microsecond precision. Industrial open-source 24 V platforms attempt to address this but introduce severe flaws. Single-board computers like the PiPLC [57] rely on operating systems, where Linux context switching introduces packet-colliding timing jitter. DIN-rail controllers like the Inoa Satellite [58] rely on a single ESP32 for both polling and intensive Wi-Fi routing, creating asynchronous blocking delays, while 8-bit shields like the AST-CAN485 [59] provide voltage access but lack multi-core redundancy. Avoiding vendor lock-in necessitates absolute timing stability. On single-core loggers, Modbus requests block the processor, disrupting real-time PWM signals and causing robotic kinematic control loops to stutter. Dedicated platforms like the FreeStation ESPiezometer [60] suffer this identical single-core blocking constraint. Furthermore, hardware translators like the EnviroDIY Modbus Wing [61] are limited to under 150 mA, rendering them impractical for robotic integration.

Marine advanced architectural designs such as the bristlemouth system [62], deliver both power and data at once over a single pair of 2-wire SPE. The Mote is an integration that converts existing RS485 input into a switched (packet), IPv6/UDP based network with the ability to route payloads from the mote to top-side gateway devices, such as spotter buoys. Although outstanding for its scalable networking capabilities, the simultaneous bandwidth usage of this encapsulated design removes all Modbus RTU timing deterministic requirements. Since legacy slaves require silent time intervals to perform their functions correctly, they can no longer be guaranteed at the micro-second level. Additionally, although the dev kit has adjustable rail voltage settings for 19 V. The smart moorings have electronic restrictions on the sustainable power output to 10 W. This budget supports API-controlled sensor power switching; however, due to the extremely large amount of surge current associated with direct-drive marine thrusters, the smart moorings will immediately blow their fuse if they try to supply this type of load. Therefore, the addition of an external trickle-charged battery buffer would be needed to provide sufficient surge protection for heavy kinematic loads. Alternatively, high voltage boards that are designed specifically for automotive applications (e.g., eChook BMS [63]) or grid-scale applications (e.g., Pionix Yeti [64]) eliminate timing jitter issues through hardware UART auto-directing, but their architectures are optimized for heavy grid contactors rather than marine PWM robotics.

The OpenWater Hub is designed to close the basic divide between purely observational systems that observe the environment and active mechatronic systems that are able to interact with their environment. While this hub allows for the extraction of RS485 industrial sensor signals out of closed commercial infrastructures (Table 1), it advances beyond passive open-source loggers (Table 2) by offering a deterministic, multi-processor node capable of simultaneously managing a 24-V RS485 network of sensors and controlling actuated components on marine robots. Thus, the OpenWater Hub connects these two disparate fields through the ability to offer four deployment methods from a single printed circuit board (PCB), all supported by completely reproducible hardware, firmware, and mechanical designs. From a hardware perspective, the 24-volt fuse-protected bus supplies up to 24 watts of power to support the high surge current requirements of optical sonde wiper motors. Users wishing to achieve zero-quiescent sleep states on the 24-volt bus, while maintaining a simple PCB design, may add commercially available optocoupled high-side P-channel MOSFET modules to physically disable sensor power. This overcomes the severe limitations of standard 5-volt data loggers, providing researchers with a much greater degree of flexibility. Separate from the sensor bus, four independent voltage step-down regulators provide 3 amps of adjustable 8.85 V DC power for the four PWM actuation channels. Because the PWM output and MCU switch pins operate as logic outputs, they can directly control brushless DC electronic speed controllers (BLDC ESCs) or independently activate external N-Channel MOSFETs to drive heavy DC water pumps and hydroponic dosing valves, thereby providing an inexpensive method of adding functionality without needing costly proprietary extension modules. Additionally, a dual-MCU architecture allows for deterministic processing. Two ATmega128A microcontrollers are employed. MCU1 processes real-time actuator control, while MCU2 performs Modbus RS485 polling, CRC16 checksums, and transceiver timing in a non-interruptible bare-metal loop. Fig. 1 shows how you can use OpenWater as an application in several configurations:

1. **Standalone Data Logger (Case Zero):** The fully populated bare PCB functioning as a test bench instrument for sensor calibration, dual-MCU validation, and robotic PWM bench testing.
2. **“Lab-in-a-Box” (Case C):** Portable, self-contained instrument for quick on-site water quality testing by a person working from a small boat or at the shore.
3. **Floating Water Sensing Station (Case A):** Autonomous, solar-powered platform that collects and transmits environmental data.
4. **Robotic Support Hub (Case B):** A command relay and power distribution center. The robotic support hub sends and receives data over an RS485 serial communication link across large distances with the intelligent payloads and directly controls soft robotic actuators using open-loop PWM (pulse width modulation) through tethered connections.

**Table 1:** Architectural comparison of commercial telemetry and sonde ecosystems.

Manufacturer/System	Primary deployment focus	Logger telemetry infrastructure	Sensor power management	High-current motor actuation
OpenWater Hub (This Work)	Water Quality & Robotic Support	Modular UART Sockets (LoRa, Wi-Fi, cellular, RF)	Off-the-Shelf P-channel MOSFET (24 V Cut)	4x Adjustable (8.85 V) / 3.0 A PWM
NexSens X-Series [31], [32]	Deep Marine Observatories	Proprietary WQData LIVE Dashboard	Native 12 V Switched Channels	12 V Proprietary Relays Only
ReNile iRoots [37]	Hydroponics & Smart Ag.	Proprietary Cloud (Data Export Limitations)	Undisclosed	Requires Standalone 5-Relay Node
YSI EXO Ecosystem [29]	Regulatory Water Quality	Proprietary KorEXO Software	Native Internal Sonde Sleep	None (Passive Data Aggregation)
Yosemitech Sondes [30]	Marine & Aquaculture	Relies on 3rd-Party Data Loggers	Sonde Controlled	None (Passive Sensing)
Aqualabo ODEON [65]	Wastewater & Industrial	Proprietary Handhelds	Logger Controlled	None (Passive Sensing)
In-Situ VuLink [34]	Groundwater/Wells	Proprietary HydroVu Dashboard	Native Telemetry Sleep Cycles	None (Passive Telemetry)
Seeed SenseCAP [35]	Industrial IoT/Global	Seeed Proprietary Cloud	Logger Controlled	None (Passive Telemetry)
Digital Matter Hawk [36]	Distributed Asset Tracking	Telematics Guru Platform	Task-Based Sleep	None (Passive Telemetry)

**Table 2:** Architectural comparison of open-source hardware platforms.

Platform	Max sensor power output	Sensor sleep mechanism	Modbus timing reliability	High-current actuation	Telemetry architecture
OpenWater Hub (This Work)	24 V Pass-Through (24 W Max)	MCU Deep-Sleep + Ext. P-MOSFET	Independent MCU Bare-Metal Polling	4x PWM (Adj. 8.85 V, 3 A) / Logic for ESCs & Relays	Modular UART Sockets (LoRa, Cell, Wi-Fi)
ALog [41]	Up to 5 V Only	Physically Cuts 3.3 V/5 V Power	N/A (Lacks RS485 Hardware)	None	None (Local SD Card)
GatorByte [42]	Up to 5 V Only	Software Sleep Only (I^2^C)	N/A (Lacks RS485 Hardware)	None	Fixed Cellular (NB-IoT/LTE-M)
OpenMetBuoy [40]	3.3 V Only	Software Sleep Only (Qwiic/I^2^C)	N/A (Lacks RS485 Hardware)	None	Fixed Iridium SBD
microSWIFT [45]	3.3 V Only	External Magnetic Hardware Switch	N/A (Lacks RS485 Hardware)	None	Fixed Iridium SBD
LOOM (Hypnos) [43]	Up to 5 V Only	Physically Cuts 3.3 V/5 V Power	N/A (Lacks RS485 Hardware)	Low-Current Relays Only	Modular Sockets (Cellular, Wi-Fi)
VSB [46]	3.3 V Only	None (Continuous Battery Drain)	N/A (Lacks RS485 Hardware)	None	None (Local SD Card)
GPSB [47]	5 V Only	Physically Cuts 5 V Power (555 Timer)	N/A (Lacks RS485 Hardware)	None	Fixed Cellular (3G GSM)
XFZ Buoy [48]	3.3 V Only	Software Sleep Only (I^2^C)	N/A (Lacks RS485 Hardware)	None	Fixed Iridium SBD
Bristlemouth (Smart Mooring) [66]	24 V Backbone (300 W Max)	Native API Port Power Cut	Non-Deterministic (Packetized)	Aux. Battery Buffer Required	Topside Gateway (Cellular/Iridium)
Bristlemouth (Dev Kit/Mote) [62]	Up to 19 V Selectable Rails	Controlled via Mooring Node	Tunneled (Lost Microsecond Timing)	<10 W Limit (Fuses Trip on Motors)	Single Pair Ethernet (SPE)
EnviroDIY + Wing [61], [67]	11.3 V Boost (100 mA Max Limit)	Physically Cuts 11.3 V Power	Single-Core Blocking Latency	<100 mA Limit (Fuses Trip)	Modular Sockets (Cellular/Wi-Fi)
FreeStation ESPiezometer [60]	12 V Boost	Software Sleep Only	Single-Core Blocking Latency	12 V Low-Current Only	Integrated Wi-Fi/BLE 5.0
Chrismettal/PiPLC [57]	24 V Pass-Through	External Relays Required	High Latency (Linux OS Jitter)	External Relays Only	Native Ethernet/Wi-Fi
eChook BMS [63]	5 V/12 V Outputs	12 V High-Side Drivers	Hardware UART Auto-Direction	12 V 5 A High-Side Drivers	CAN Bus/SD Card
Pionix Yeti [64]	12 V Output (from AC Mains)	Grid Contactor Logic	Hardware UART Auto-Direction	AC Grid Contactors	Ethernet/MQTT (via Yak)

## 2 Hardware description

The OpenWater Hub has addressed the current lack of standardization and modularity with regard to hardware configurations in both marine robotics and environmental sensing. Usually, when moving from a benchtop prototype testing environment to a field-portable testing environment and then to a long-term offshore monitoring system, developers are required to design or develop entirely different electronic systems. This development results in increased development times and makes maintenance much more difficult. We have resolved this problem by developing a single, open-source printed circuit board (PCB) that includes all the necessary subsystems for computing, communications, and power management.

The PCB serves as the primary hardware core unit that is capable of being used in at least 4 different operational configurations. Case Zero refers to the basic hardware configuration used for calibration and data collection. Case A uses the hardware in a floating sensing station operating autonomously. Case B extends the functionality of Case A by using the hardware as an autonomous robotic support hub. Case C uses the hardware as a portable laboratory that can be held in one’s hand. Fig. 1 illustrates a conceptual representation of how the unified architecture can adapt to the multiple marine environments.

**Fig. 1:**
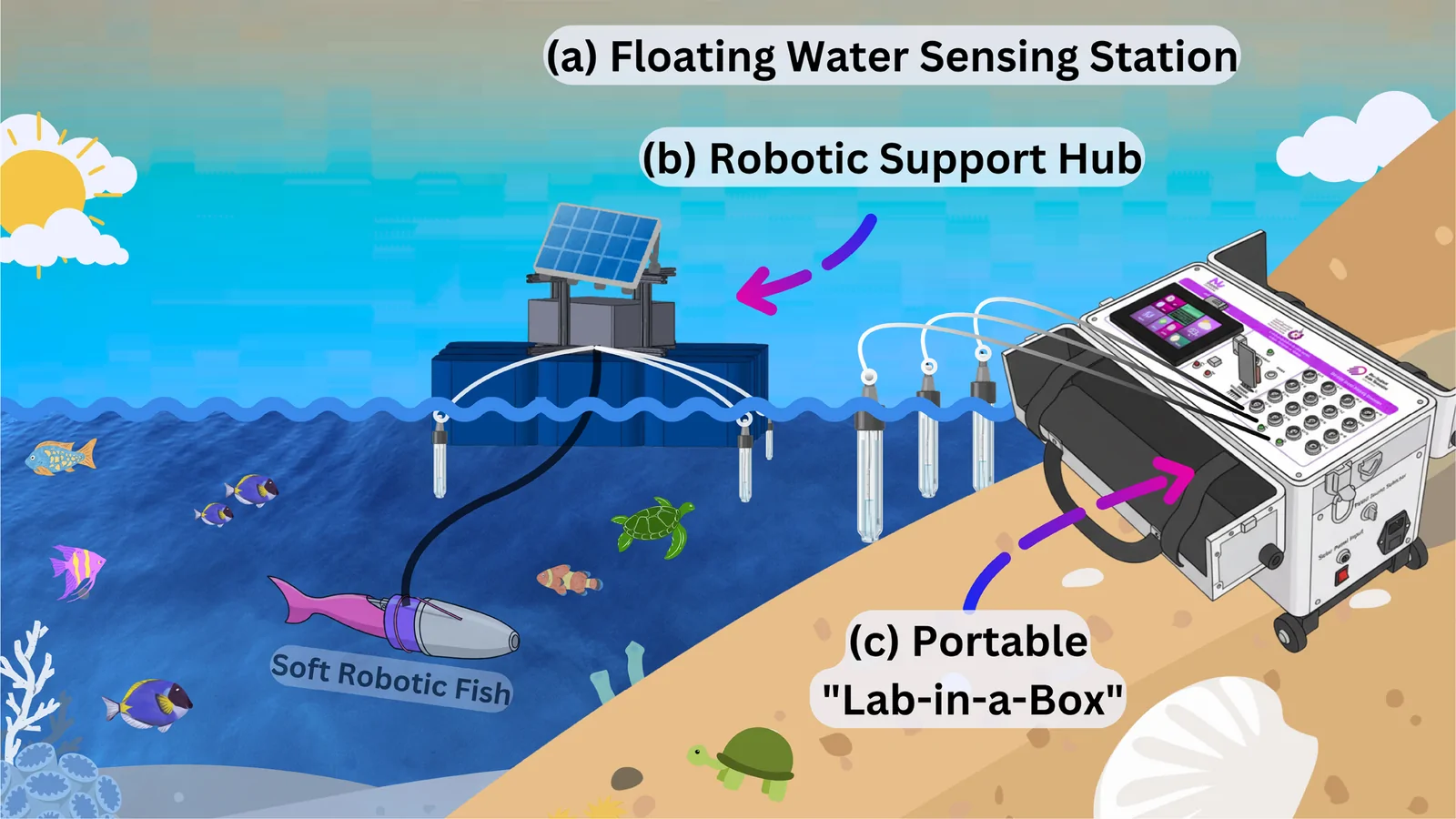
**Conceptual Overview of the OpenWater Hub’s Three Operational Configurations. (a)** A floating water sensing station, with its own array of water quality sensors, and **(b)** a robotic support hub, providing power and data to a tethered underwater robot. The third configuration, **(c)** the portable “Lab-in-a-Box”, is depicted as a separate, mobile unit ready for rapid field deployment.

### 2.1 The core hardware: OpenWater hub PCB architecture

Rather than follow conventional hierarchical primary/secondary processing architectures, the design intentionally separates two ATmega128A microcontrollers in order to functionally isolate them. They each operate independently and are physically separated from one another with respect to their areas of operation. As such, neither the time spent on sensor acquisition nor communication will ever interfere with motion kinematics.

#### 2.1.1 Dual-microcontroller processing logic

The design purposefully rejects the traditional hierarchical structure of primary/ secondary processing. Instead, there are two equal, physically separated microcontrollers that have the same functionality in each domain. This separation prevents any delay from sensor polling to interfere with motor kinematic processing.

• **Actuation microcontroller (MCU1):** Dedicated solely to physical mechanics, this microcontroller controls all PWM signals used to drive the motor regulators and manages the physical states of the attached robotics.
• **RS485 sensor microcontroller (MCU2):** This processor is a stand-alone digital data hub. In addition to being responsible for managing the time-consuming Modbus RTU polling sequences, the processor is also responsible for parsing raw byte streams from industrial probes and validating data integrity through local CRC16 checksums prior to routing.

We chose the ATmega128A over traditional 8-bit options such as ATmega328 because of its dual hardware serial ports. Two separate hardware UART interfaces are required for simultaneous Modbus RTU communications and external telemetry. Modern ARM Cortex processors were skipped to minimize overhead. Non-blocking serial comms on a single-core ARM would normally require a real-time operating system. The modular nature of the dual-MCU 8-bit design provides for modularity. Separating the Modbus protocol to a dedicated co-processor also prevents polling latencies from interrupting the actuation MCU’s motor control loops. This topological arrangement allows users to completely customize their firmware using an accessible Arduino IDE.

The PCB has no data-carrying copper traces connecting the two MCUs. This separation will prevent high-frequency switching noise produced by the motor regulators from affecting serial communications. There are two ways that researchers/developers may create a method of communication between the two MCUs. The primary method is to utilize two LoRa modules mounted on headers H3 & H4 to form an internal wireless mesh. MCU2 sends all parsed sensor telemetry data; MCU1 receives this telemetry data via the air interface (wirelessly) just inches away and converts this data into kinetic action if necessary. If researchers wish to use a wired connection, they may connect a jumper wire between the isolated UARTs on programming header H1/H2 (Fig. 2).

**Fig. 2:**
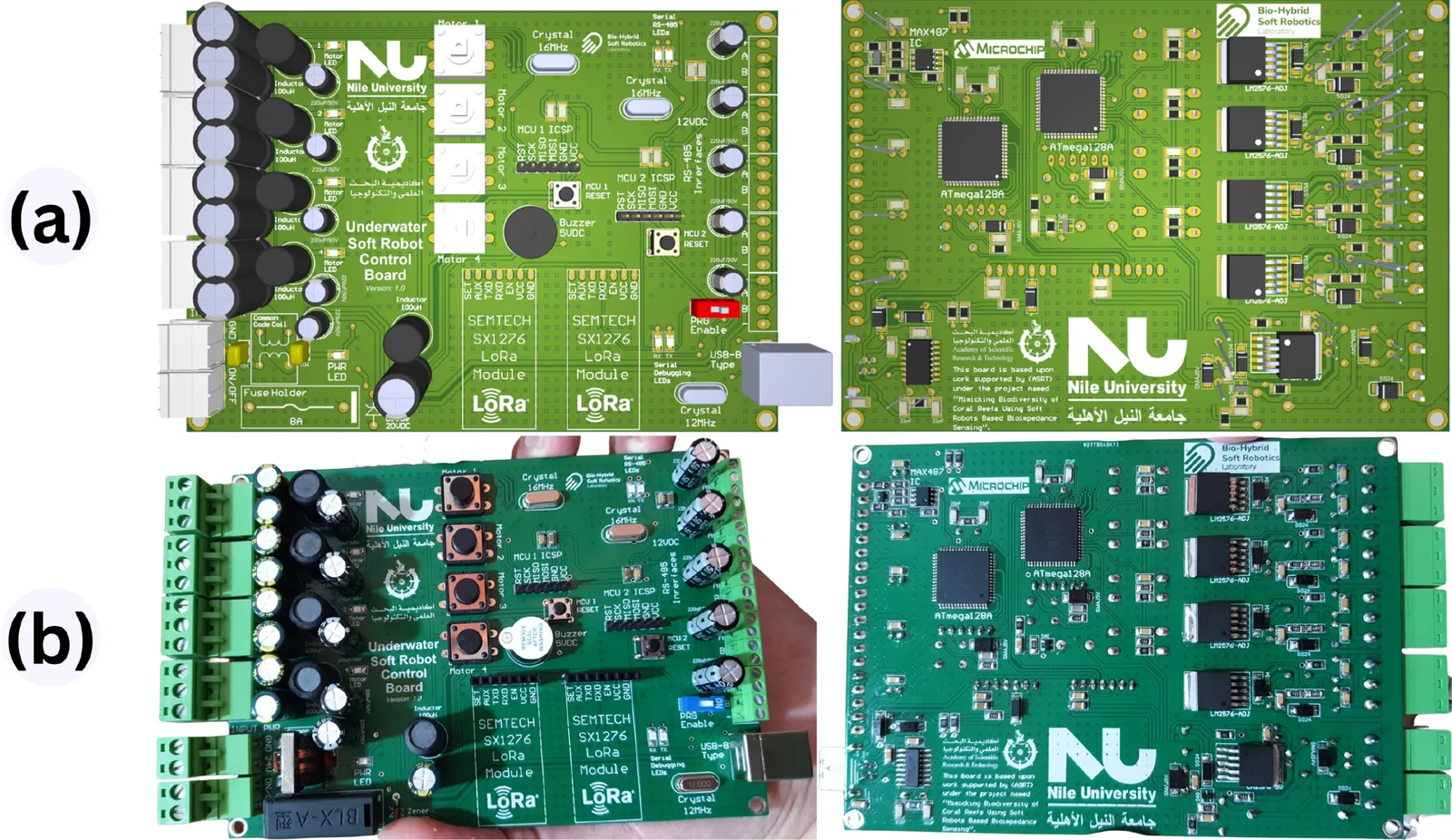
**The OpenWater Hub PCB Assembly. (a)** The CAD rendering of the populated board showing component placement. **(b)** The physical board after fabrication and assembly. Key components include the terminal blocks for sensor wiring (left), the dual MCU chips (center right). The board measures 143.51mm×102.235mm and common RC servos or raise.

#### 2.1.2 Communication and sensor interfaces

The PCB integrates specific interfaces required for marine applications, moving beyond standard TTL serial connections to robust industrial protocols.

**RS485 Sensor Bus:** The MAX487 transceiver IC connects to a half-duplex RS485 bus at the main terminals; this connection permits the OpenWater Hub to interact with industrial water quality sensors in the field using cables that exceed 50 m. Because the differential signal used in this system rejects common-mode noise that is picked up by the longer tethers in the conductive seawater environment, no additional noise reduction circuitry was implemented into this design. The OpenWater Hub is also manufacturer agnostic. Any new sensor can be integrated onto the hub if its compliance with the baseline electrical (RS485 differential signaling) and communication requirements are met. Specifically for compatibility purposes, a third-party sensor must meet all three requirements: RS485 differential signaling (hardware), Modbus RTU protocol formatting (software), and acceptance of 12 VDC to 24 VDC input voltage tolerance (power). Once these minimum requirements have been met, there is nothing stopping the researcher from deploying that sensor on their system. At that point, the processor simply reads off the calculated memory register values provided by each individual sensor. This capability of accepting many different types of probes has allowed researchers to take advantage of specialized industrial equipment such as Pyxis Lab inline fluorometers [68] but has also permitted the system to accept low-cost, value-tier environmental probes. In addition to high-end environmental probes, compatible alternative probes natively support these parameters and therefore may be utilized in conjunction with the OpenWater Hub, including Yosemitech Sondes [30], Rika Sensors [69], Jinan Renke water quality transmitters [70], Seeed Studio Environmental Sensors [71], and DFRobot industrial probes [72]. The measurement accuracy and operating range of each probe depend entirely on the probe itself. For example, in this study, JXCT probes were used to collect data. As a baseline for operation, the JXCT pH sensor measures 0 to 14 pH with an accuracy of ±0.5 pH [73]. The temperature transmitter operates over a range of −55 °C to +125°C with an accuracy of ±0.5°C

**Wireless Expansion Sockets:** Wireless expansion sockets are available on the circuit board, which have 2 programmable socket headers that map to the UART hardware port of each MCU. A variety of wireless telemetry modules can be used with these sockets. The system was primarily evaluated using a DRF1278DX LoRa module (433 MHz, 500 mW) and AS32-TTL-100 LoRa module (433 MHz,100 mW). The footprint of the evaluation module is compatible with other UART-based radios, including nRF24L01 + adapters via UART, ESP8266/ESP32 WiFi modules. This will allow researchers to match the link budget and bandwidth requirements to their individual deployments. Fig. 3 illustrates the signal path and location of specific interfaces to enable this modularity. Additionally, while the use of UART limits access to physical layer diagnostic information such as RSSI/SNR, researchers who require additional link budget tracking may replace the standard radio module with an SPI-based module or an ESP32 microcontroller.

**Configurable Inputs:** The board has four tactile push buttons (SW2-SW5), that connect to MCU1. They provide logical input to allow for HMI interaction with software. An operator may be able to make one of the buttons switch modes (i.e., calibration mode and deployment mode). There will be no need to reflash the firmware for any button. One of the switches on the board (SW1), is a dedicated DIP switch that isolates the CH340 USB serial bridge from the internal UART. It provides a way to prevent data collision while an operator is updating firmware using an external programmer. (Fig. 2).

**Dual-MCU Wireless Telemetry:** To prevent bandwidth bottlenecks from occurring in telemetry data transmission through a single device, the dual-processor design includes a separate wireless module for each processor. Both processors have LoRa transceivers that are mounted with two sets of header pins (H3 & H4). Each set of headers is used by a separate processor for transmitting its own data at different frequencies if needed. Using this architecture, researchers can configure the actuator MCU to send out structural telemetry and battery state information on one frequency channel while sending sensor data related to the water quality using the RS485 sensor MCU on a second frequency channel. Conversely, if the operator prefers a common data stream and wants to be able to transfer commands or data between processors without having to use a wire-based connection, the board can be populated so that both headers are utilized, thus creating a wireless bridge that allows the two processors to communicate internally. In addition to internal routing, this wireless system also allows for the platform to function as an offshore telemetry unit which is able to connect the payload data received from submerged RS-485-based sensor arrays and tethered robotic units to the shore-side LoRaWAN gateway and a cloud-based analytics dashboard like Blynk or Arduino IOT Cloud (see Fig. 4).

**Fig. 3:**
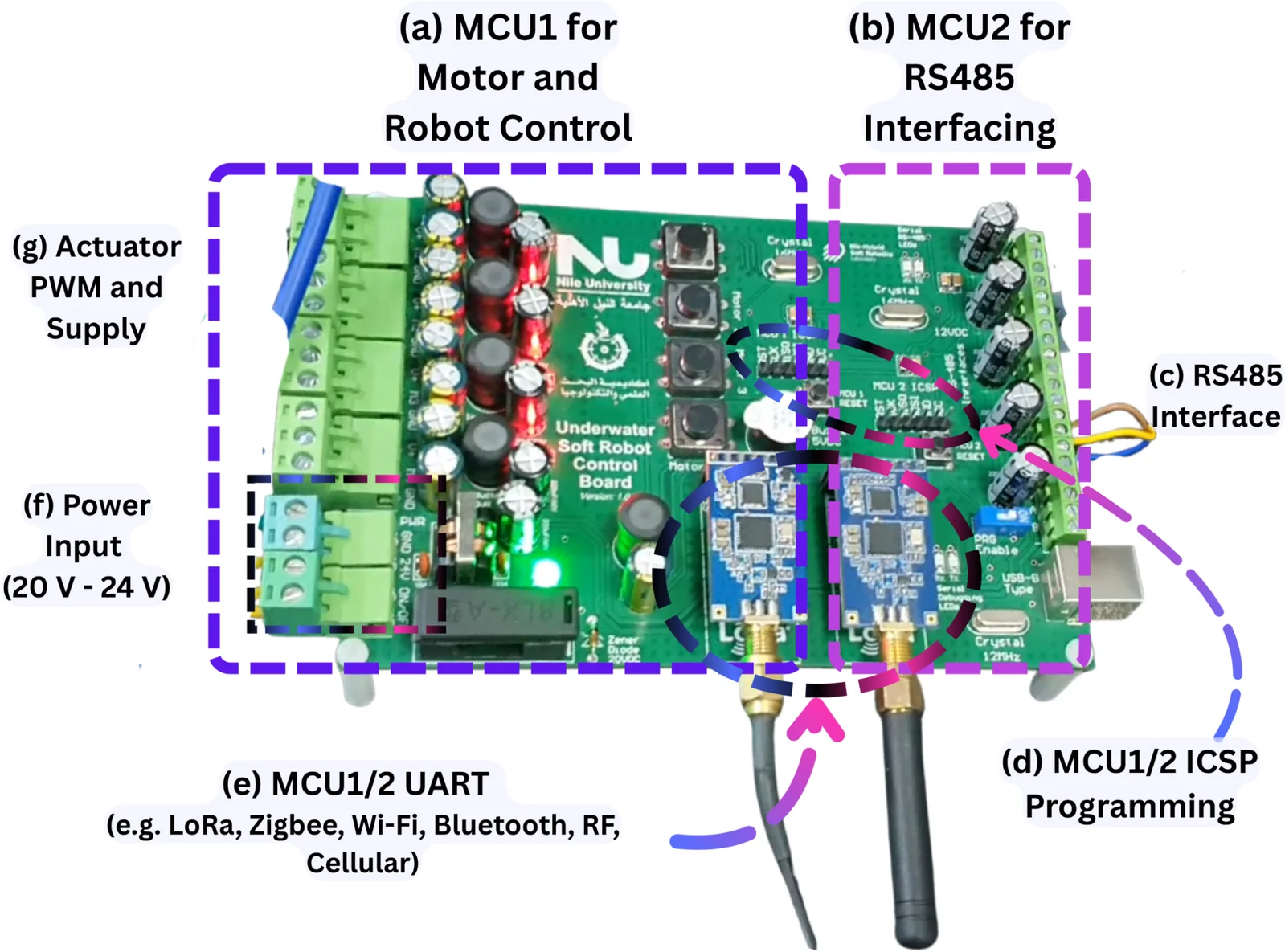
**PCB Architecture and Interface Map.** This diagram details the functional blocks of the PCB. **(a)** The MCU1 block handles motor logic and main system control. **(b)** The MCU2 block manages the RS485 sensor bus. **(c)** The RS485 interface terminal provides the physical connection for external sensor arrays. **(d)**ICSP/UART programming headers. **(e)** Dual UART sockets accommodate LoRa modules (e.g., SX1278), Zigbee, Wi-Fi, Bluetooth, cellular or any UART-based module **(f)** The Motor PWM and board supply terminals allow for actuator connection and power input.

**Fig. 4:**
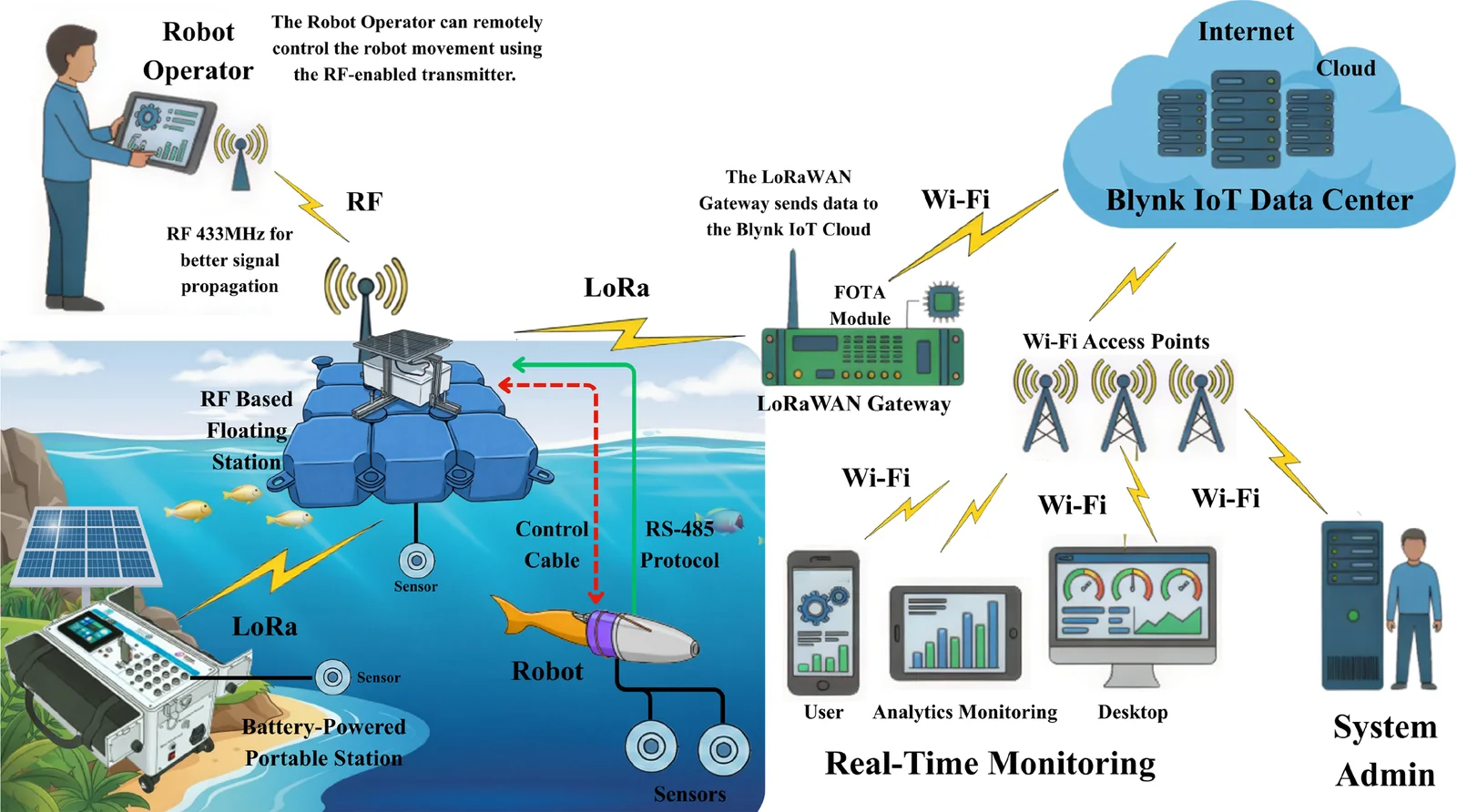
**Network Architecture and Data Flow.** This diagram illustrates the system-level connectivity facilitated by the **OpenWater Hub**. The Hub (labeled ”RF-Based Station”) takes data from submerged **RS485 sensors** and the underwater robot via a wired tether. It then uses the onboard **LoRa module** to transmit telemetry packets wirelessly to a shore-side **LoRaWAN gateway**, bridging the gap between the underwater environment and the cloud-based analytics dashboard.

#### 2.1.3 Actuation and power management

The OpenWater Hub is created to directly power electro-mechanical systems so that there is less reliance on individual breakout boards.

**Motor Control Channels:** There are four independent motor control channels which provide PWM output. The PWM output can be used with either marine servo motors or ESCs (electronic speed controllers), and therefore this board can be used to control various actuators including, but not limited to the control surface of an aquatic soft-robotic fish, or the winch mechanism in a profiling system. It can even be used with DC motors when combined with MOSFETs using the configuration detailed in Section 2.1.4.

**Power Regulation:** The design of the board has an extremely narrow and safe input voltage range of 20 V to 24 V DC (customizable) to provide long-range power delivery to the sensors and account for any possible voltage drops. Using onboard switching regulators (the LM2576 family), we have reduced these inputs into regulated power rails for both the logic circuits (5 V) and the external peripherals. While theoretically capable of handling inputs of up to 40 V, the board’s input is limited by hardware. The input protection circuit consists of two discrete stages: D7, a 24 V transient voltage suppressor (TVS) that clamps high-voltage surges, and D11, a 20 V Zener diode that functions as an under-voltage lockout (UVLO) gate to prevent the logic systems from booting under unsafe low-voltage brownout conditions. Inputs greater than 24 V will cause the TVS diode to be triggered, which will shunt the input down and prevent damage to downstream components.

#### 2.1.4 Hardware customization and system boundaries

The OpenWater Hub architecture functions as a flexible foundation that researchers can modify for highly specific deployment parameters. The board restricts total logic and RS485 sensors draw to 1.0 A through the primary fuse (F1) (at 20 V – 24 V) for protection. The actuation plane bypasses this fuse, allowing researchers to draw up to 12.0 A across the four motor headers. The LM2576-ADJ regulators governing these motor channels possess a hard physical silicon limit of 3.0 A per channel. If a connected actuator draws excessive current, the regulators will trigger internal thermal protection to prevent component failure. Replacing these regulators with higher-capacity equivalents is not supported, as pushing continuous currents beyond 3.0 A could induce magnetic saturation in the 100μH inductors and destroy the SS34 Schottky flyback diodes.

Operators are able to modify the preset 8.85 V default motor output based upon their needs. Hardware design utilizes a simple resistor divider circuit that determines this default value. Users of the system may choose to replace the 62 kΩ “top” resistor (denoted by $R_{\text{top}}$) on any individual motor channel. Users can then select an appropriate target voltage using the standard reference equation below (Eq. (1)):

(1) $$V_{\text{out}}=1.23\;\text{V}\times \left(1+\frac{R_{\text{top}}}{R_{\text{bot}}}\right)$$

Where $R_{\text{bot}}$ will always be set to 10 kΩ. Thus, users may drop particular channels to 5 V for common RC servos or raise selected channels up to 12 V to support larger industrial-type actuators.

The baseline power architecture is set at a 24 V level, which is controlled by a 24 V transient voltage suppressor (D7) and a 20 V Zener diode (D11), as the gate for the under-voltage lockout (UVLO) circuit. For deployments using a standard 12 V marine battery, D11 can be desoldered and replaced with a 9 V or 10 V Zener to lower the boot threshold.

To eliminate idle power draw from permanently active RS485 sensors, users can insert a P-channel MOSFET module with an optocoupler (e.g., HW-548) directly into the 24 V sensor supply wire. This module could be controlled by the PB1 pin on the RS485 sensor MCU to cut off power to the probes without the danger of ground loops that could destroy the RS485 bus.

Continuous field deployments of RS485 sensors require a baseline quiescent current. The native board layout sends the 24 V to pins J5-J9. Users needing longer battery life can tap this supply rail. Connecting a high-side P-channel MOSFET in the positive 24 V going to the sensor array gives you a physical power gate. This structure is detailed by Fig. 5. The gate of this external device is connected to pin 5 (PB1) at the MCU2 ICSP header (h1). When toggling PB1, it will allow the MCU2 processor to shut off sensor power when sleeping. High-side switching is a strict physical necessity. Using a low-side N-channel MOSFET would sever the ground reference and make the 24 V return path go out the RS485 A & B lines and kill the transceiver.

**Fig. 5:**
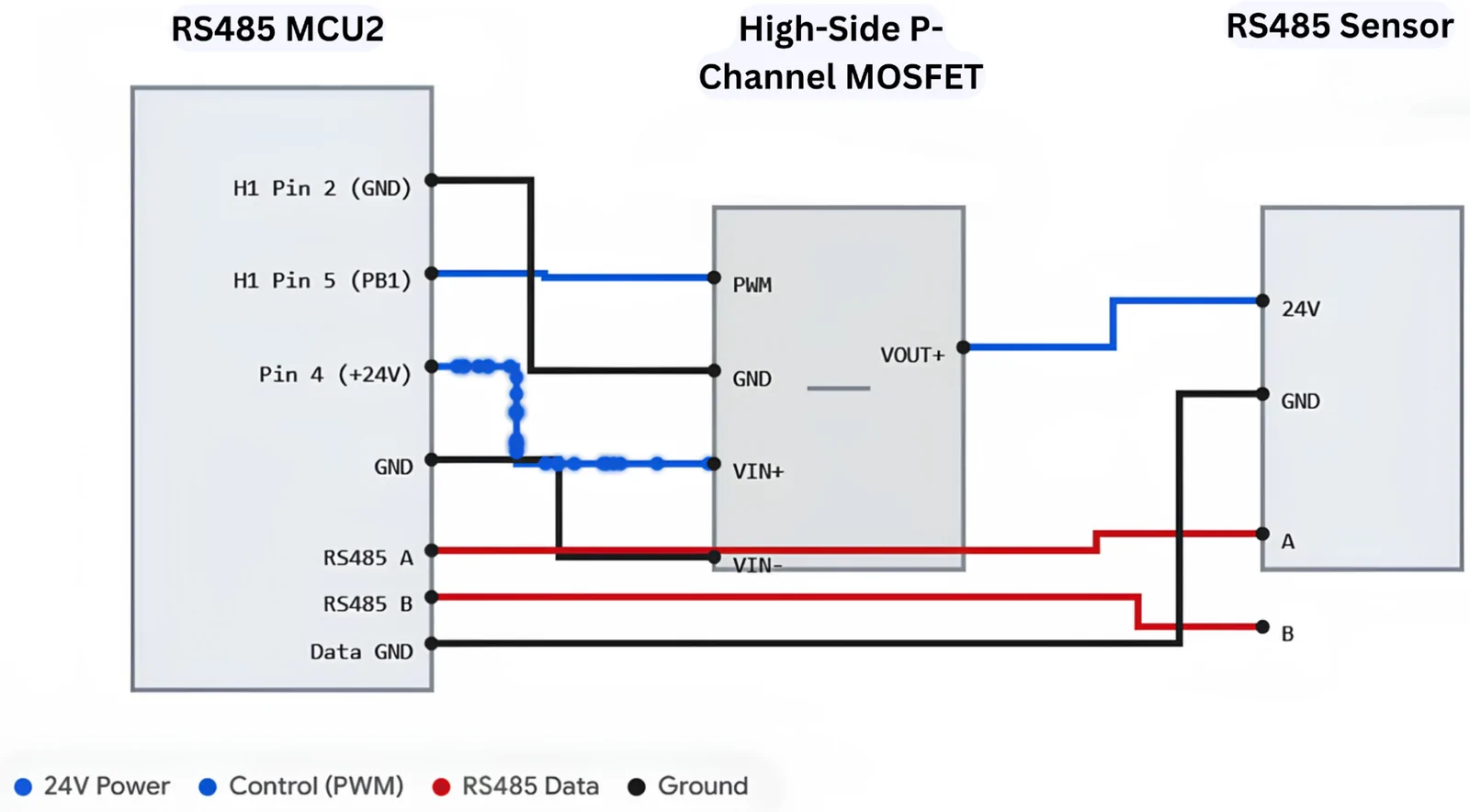
**High-side power MOSFET gating for RS485 sensors** This configuration reduced idle power draw for sensors.

### 2.2 Base configuration: Standalone data logger (Case Zero)

The fundamental operational mode of the OpenWater Hub is the **standalone data logger (Case Zero)** shown in Fig. 6. This configuration operates the fully populated PCB on a test bench without complex environmental enclosures. Rather than just a static testing baseline, researchers could use this fully populated state as a completely open sandbox to build whatever they want. They can test complex robotic kinematics, drive heavy motors, calibrate sensors, and experiment with dual-MCU serial routing all simultaneously. As a matter of fact, the subsequent field deployments (Case A and Case B) operate directly on this fully populated foundation, though operators could easily strip them down to semi-populated variants based entirely on what their specific field mission needs.

In this configuration, the hardware setup involves:

1. **Power Supply:** The board uses a 24 V input for powering the board. For laboratory testing, you will use a benchtop DC power supply (or a wall adapter) to power the board. For field testing/prototyping, you can use a rechargeable lead–acid sealed battery that has been charged to 12 V, or a solar cell output. This power source then goes into a DC-DC step-up converter so it puts out a 24 V bus voltage.
2. **Sensor Connectivity:** This system is RS-485 protocol-agnostic. Any sensor that communicates with an industry-standard Modbus RS485 framework can be connected. Researchers do not have to purchase from one company. In this research, we used industrial JXCT water quality sensors. We had them connected by wiring into the **RS485 screw terminals**.
3. **Data Logging and Visualization:** The board will send the sensor data that has been parsed to a location where it can be viewed as soon as it is received. It may also stream this data to a remote gateway using its onboard **LoRa module**. Additionally, the board may stream this data immediately to a connected laptop by sending it through the serial interface for both immediate analysis of the sensors and calibration of them.
4. **Actuator Testing:** In the case of the fully populated board within the sandbox configuration, the large motor regulators along with the first actuation MCU1 are 100% functional. Therefore, researchers may be able to attach heavy actuators, test the kinematic characteristics of actuators, and perform research using actual robotic devices directly at their workbench prior to being required to build a floating chassis (Fig. 6).

**Fig. 6:**
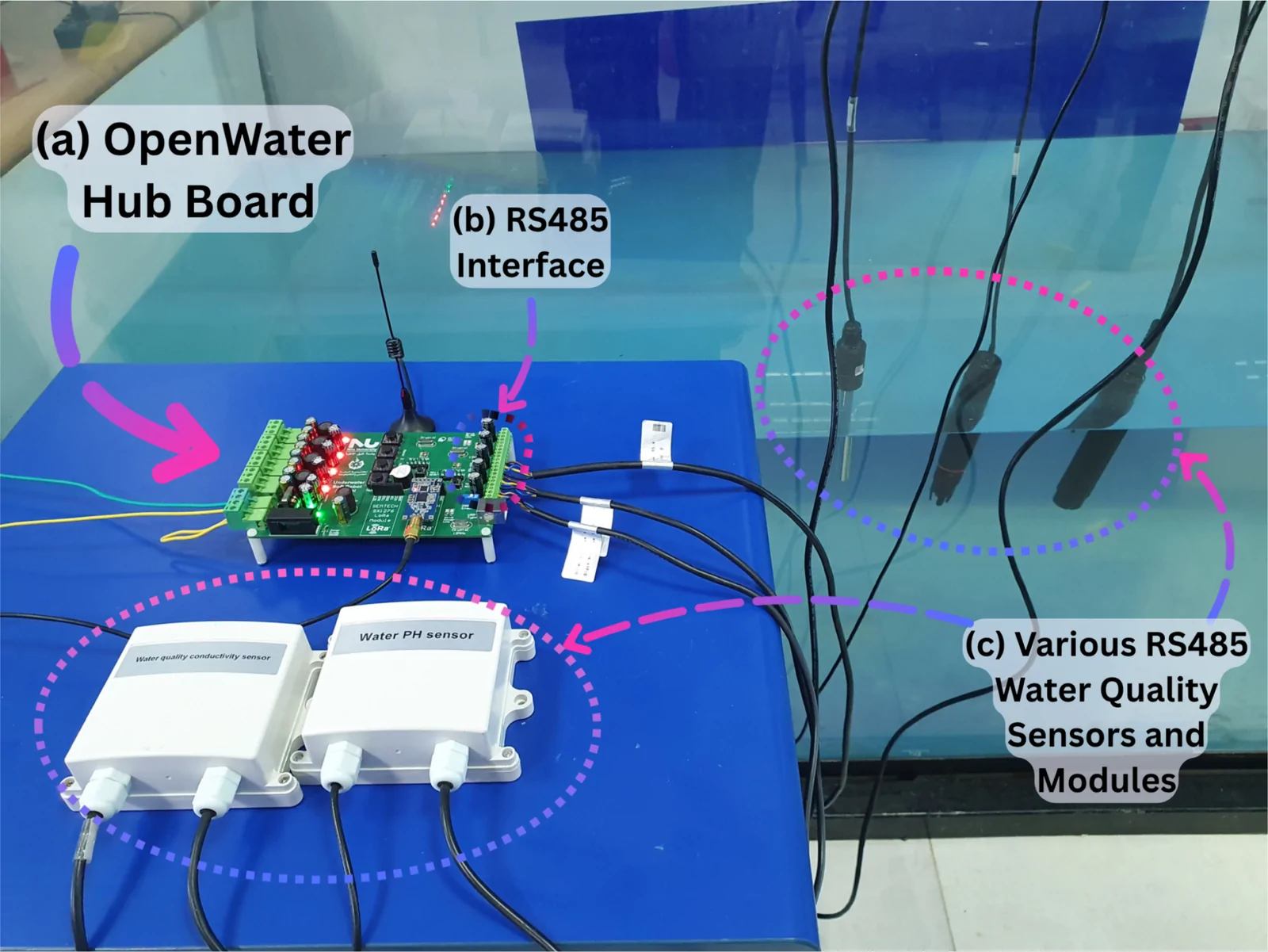
**Validation Setup for Case Zero.** This figure demonstrates the **(a)** OpenWater Hub functioning as a standalone logger. The standalone PCB is powered on a test bench and connected via the **(b)** RS485 bus to **(c)** three industrial water quality probes submerged in a test tank.

### 2.3 Implementation 1: The portable “Lab-in-a-Box” (Case C)

Case Zero’s benchtop data logging system will provide enough functionality to perform the initial calibrations required to determine the accuracy of sensors; however, all field applications will need a hardened version of the equipment that can withstand harsh environmental conditions. Additionally, since these units will be deployed off-grid or at least for longer than would typically be acceptable on an electrical outlet, they will also require battery power. The solution to both of these challenges will be addressed by transforming the OpenWater Hub PCB into a self-contained, transportable field lab (Case C). The details of this field lab’s design can be seen in Fig. 7. In this format, the individual board becomes a handheld unit much like commercially available water quality measurement devices. However, unlike their commercial counterparts, it will have an open hardware platform for modification as well as allow users to add additional modules/sensors.

#### 2.3.1 Mechanical enclosure and HMI design

An **EGA Master 51012 Aluminium Tool Case** (or any cheaper similar generic LxWxH $455\times 205\times 320\;{\text{mm}}^{3}$) houses the system, chosen for its corner reinforcement and built-in transport wheels, both of which will be important in rugged coastal deployments. The main interface for the user (human-machine interface, or **HMI**) consists of a custom-made acrylic front panel.

• **Visual Interface:** A **capacitive touch screen from DWIN** displays real time visualizations of sensor readings and battery status.
• **Sensor Connectivity:** The right-hand side of the panel features a physical 4 × 4 grid of splashproof aviation connectors (**GX16-4pin**). This array provides 16 distinct ports, all wired internally to the **RS485 bus**. High-density connections allow researchers to use a large number of probes (e.g., pH, turbidity, dissolved oxygen) at once without having to add external splitters or expose the internal wiring. Industrial-type RS485 probes may also be used with longer cables. Researchers can position their sensors at specified water column heights using custom-made cables. Also shown in Fig. 7 is the conceptual design as well as the finished product after it was fabricated and assembled.

#### 2.3.2 Dual-source power management system

Energy flexibility is one of the most important demands of a field instrument. The “Lab-in-a-Box” has a hybrid power design managed through a rotating selector switch on the front panel. All the inside connections are described in Fig. 8.

1. **Grid Power Route:** The Lab-in-a-Box has an **IEC C14 connection** for standard 220 V AC line power. Once connected it sends the power to an interior industrial 24 V DC power source (PSU).
2. **Solar/Battery Route:** The Lab-in-a-Box also has a dedicated circular DC connection on its outside casing for power from an external photovoltaic panel. When connected, the solar panel input goes into a **PWM solar charge controller**. The PWM solar charge controller will regulate how much the internal 12 V 9000 mAh lead–acid battery receives.
3. **Voltage Control:** The internal 12 V battery output is boosted with a **DC-DC boost converter** to get the 24 V needed by the main system bus. We chose the 24 V – 20 V standard because many industrial RS485 sensors such as the JXCT series used in our study need a steady 12 V – 24 V supply. We have added a boost converter to provide a stable 24 V – 20 V output, regardless of the battery’s discharge curve. Therefore we ensure that all peak solar charging voltages (∼28 V in series battery configurations) will not activate the 24 V TVS protection diodes located on the board, therefore we protect our equipment at the same time we overcome the voltage loss on longer cables.

All of the wiring layout of this hybrid power structure along with its circuit breakers, and the signal path between the charge controllers and boost converters are illustrated in detail in Fig. 9.

**Fig. 7:**
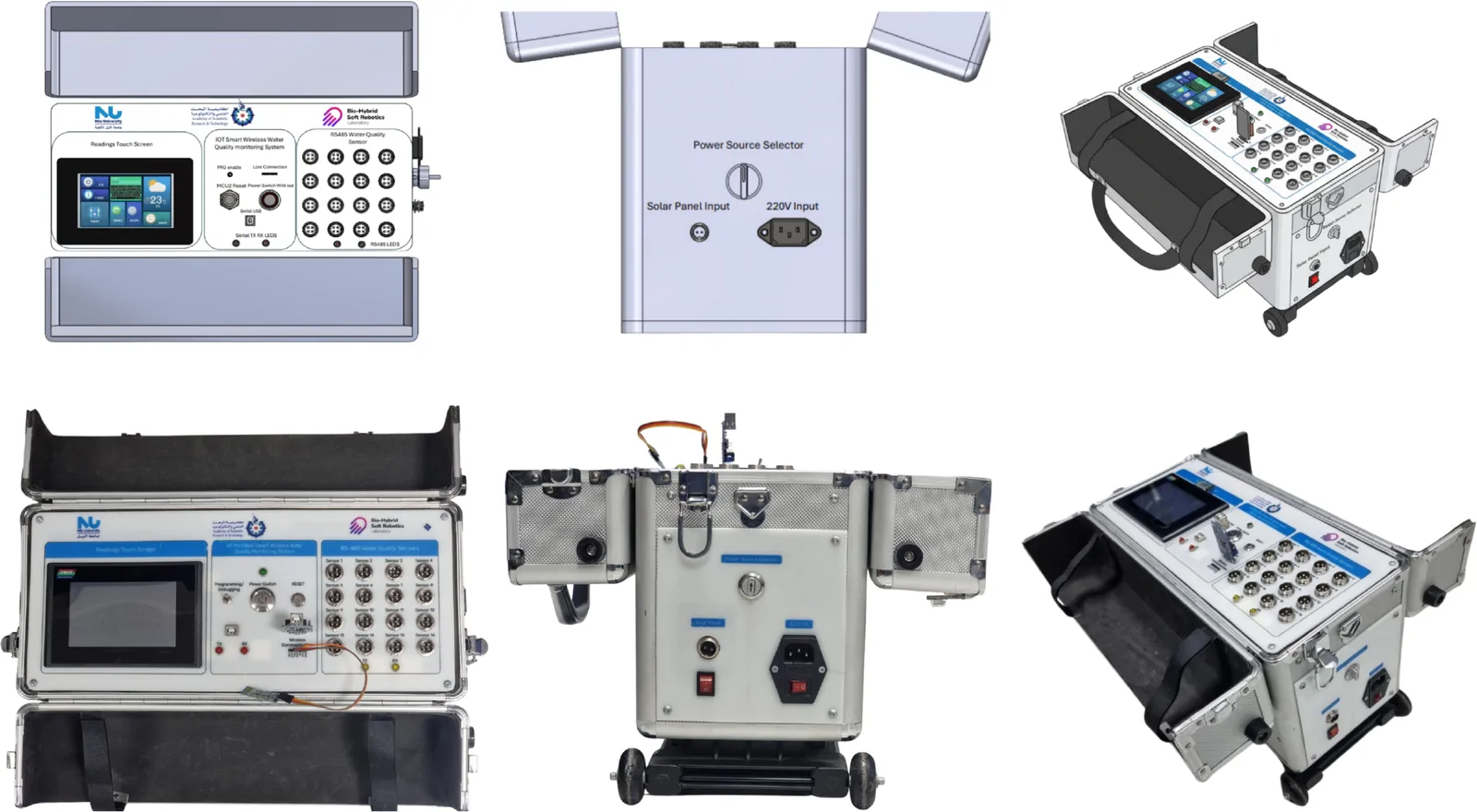
**Conceptual External View.** A visualization of the finalized portable laboratory.

**Fig. 8:**
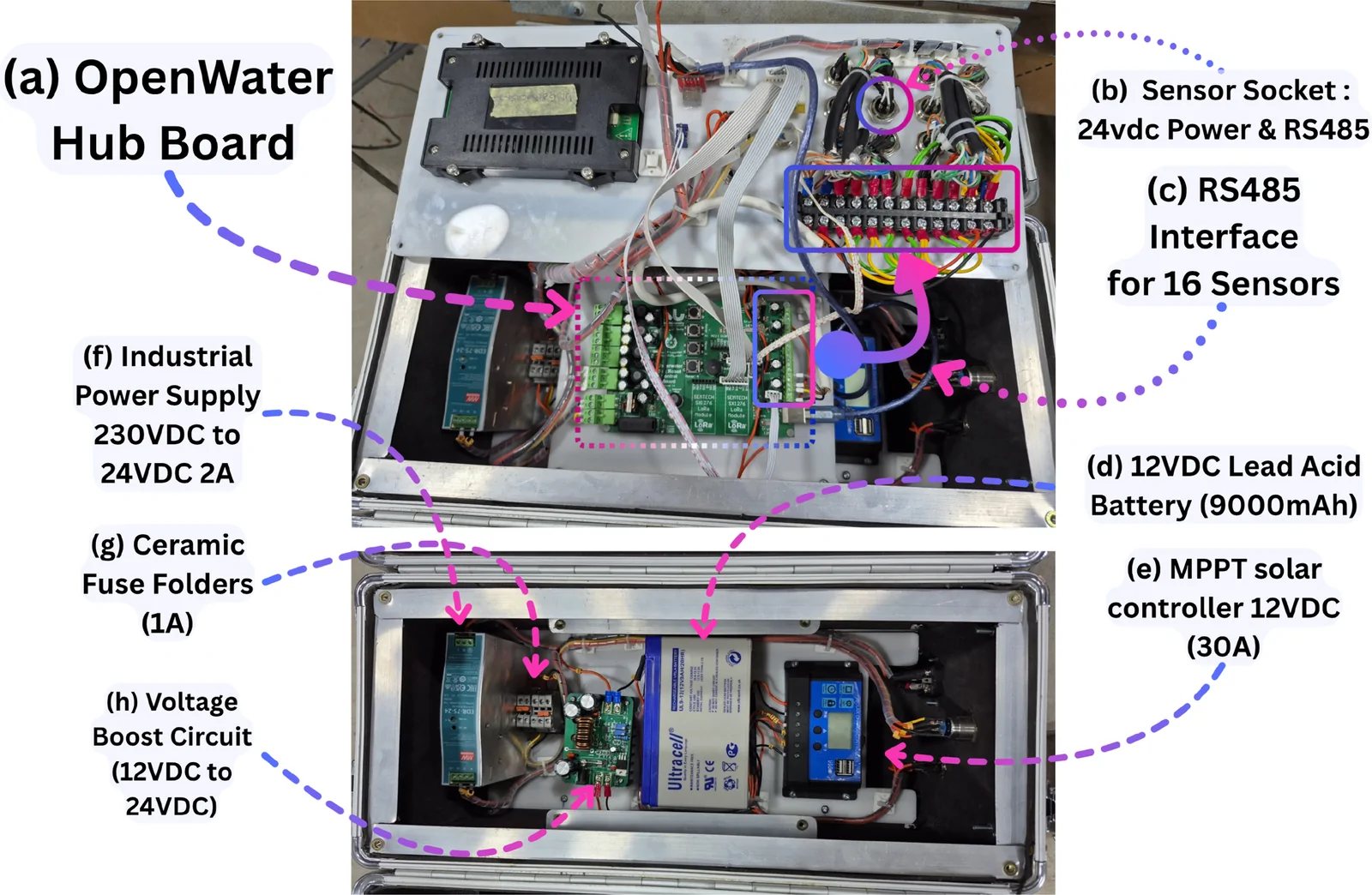
**Internal Architecture of the Portable Lab.** The dual-compartment chassis separates signal routing from primary power storage. (a) The OpenWater Hub board acts as the central control node. (b) 24 V DC and RS485 probe connectivity is supplied via external aviation sockets. (c) An internal distribution block bridges up to 16 external connections onto the primary RS485 bus. (d) A lead–acid battery with a capacity of 9000 mAh provides energy storage at 12 V DC. (e) A PWM charge controller manages photovoltaic inputs with a current rating of 30 A. (f) An industrial power supply delivers 24 V DC at 2 A for grid-connected operation. (g) Ceramic fuse holders with an overcurrent limit of 1 A are installed to isolate the supply lines against overcurrent events. (h) A DC-DC boost converter steps up the battery output from 12 V to the required 24 V level.

**Fig. 9:**
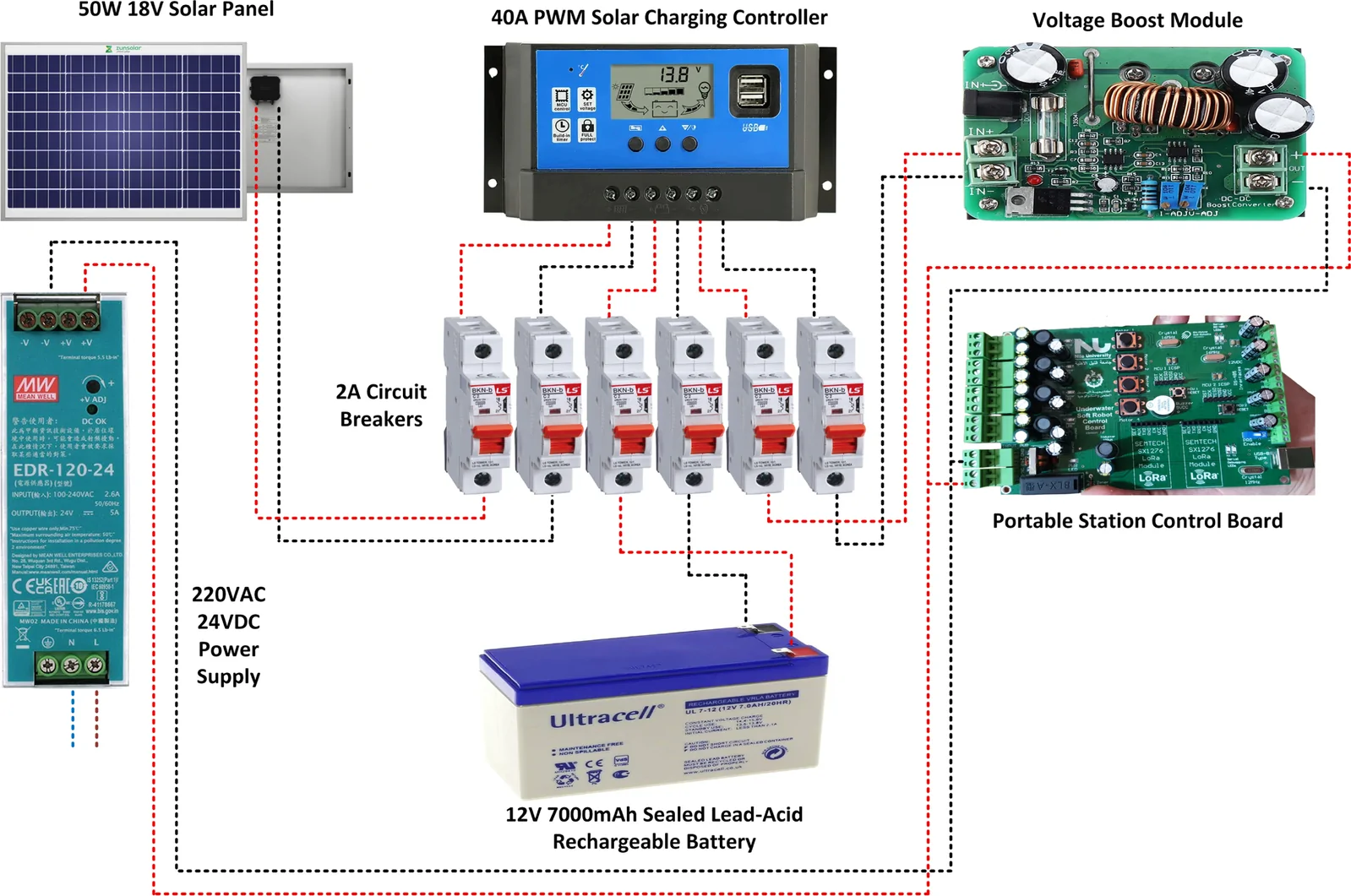
**Hybrid Power System Wiring Diagram.** This schematic illustrates the dual-source power architecture of the **portable lab**. The system accepts either 220V AC mains (converted to 24V DC) or 18V DC from a 50W solar panel (12 V nominal). A **PWM solar charge controller** manages the charging of the 12V7000mAh / 9000mAh**lead–acid battery**. A **DC-DC boost module** steps up the battery voltage to stabilize the 24V bus required by the **OpenWater Hub board**. Circuit breakers (2A) are installed on all critical power lines for overcurrent protection.

### 2.4 Implementation 2: The offshore data collection system using a modularly designed floating sensing & support station (Cases A and B)

This last implementation places the hardware offshore and has a single, self-contained unit which will be deployed as an autonomous, long-term data collection system. The design is also modularized at the structural component level. Case A can support one OpenWater Hub board while Case B can support two stacked boards.

#### 2.4.1 Floating mechanical base

As depicted in Fig. 10, Fig. 11, the base consists of nine modular HDPE pontoon cubes measuring approximately 500×500×400mm each. Each cube includes a modified internal locking pin featuring a stainless steel threaded rod; these are used to secure the other side of the cube. These threaded rods are then attached to the aluminum truss frame, which was made from 4080 and 4040 T-slot extrusions.

A key feature is the ability to keep the floating station stationary by minimizing the effects of tidal drift. As such, marine-grade eye bolts were placed at the outer corners of each cube connecting them. Rather than placing the mooring stress at a single corner where it could cause damage to the structure, the eye bolt distributes the mooring load among several flotation modules. The aluminum truss serves as a rigid mounting surface for the IP66-rated electronics housing and provides a rigid mounting point for the solar array. IP67-rated PG9 cable glands seal all exterior wire routing. Fig. 12 shows the complete mechanical base after it had been assembled and before being transported into the field.

**Fig. 10:**
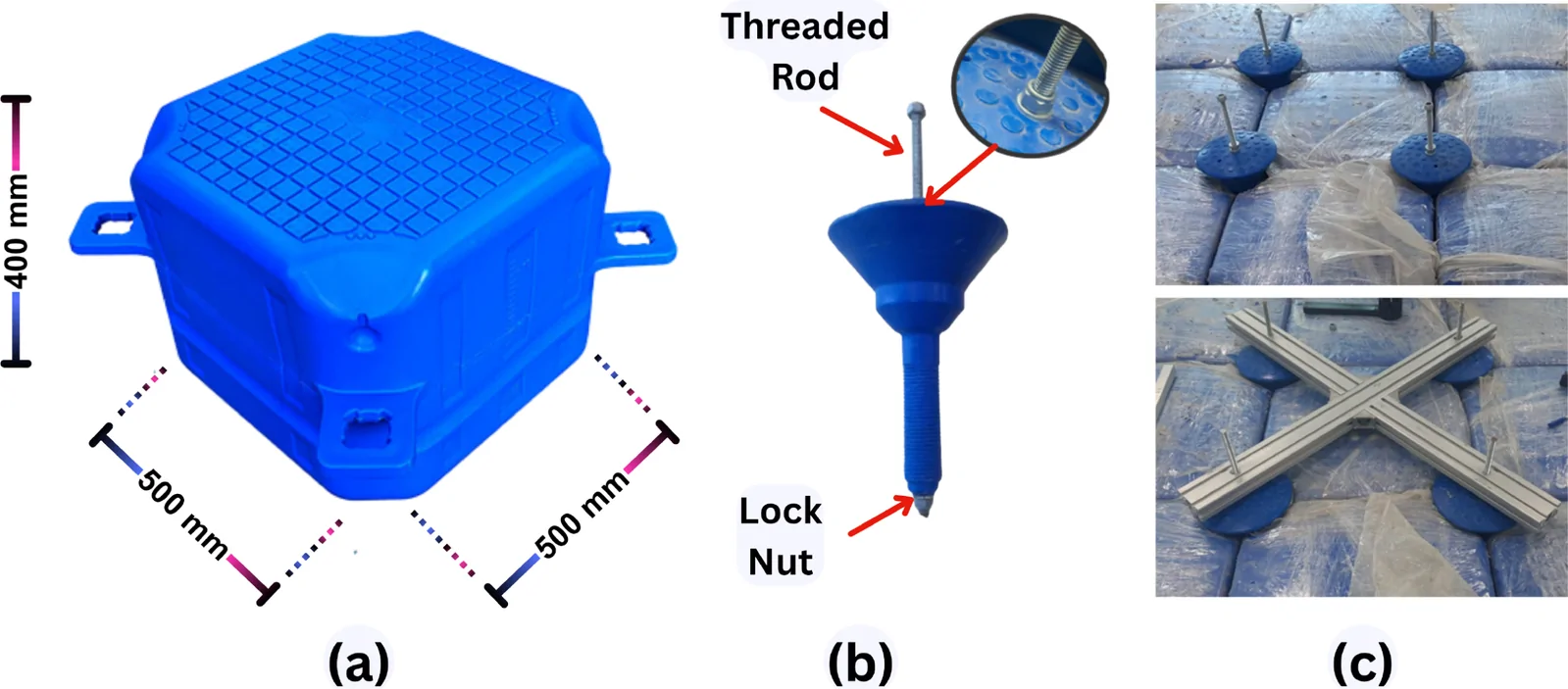
**Pontoon Base Assembly. (a)** Dimensions of the HDPE pontoon cube. **(b)** The modified locking pin with embedded threaded rod. **(c)** The aluminum base frame anchored to the pontoon junctions.

**Fig. 11:**
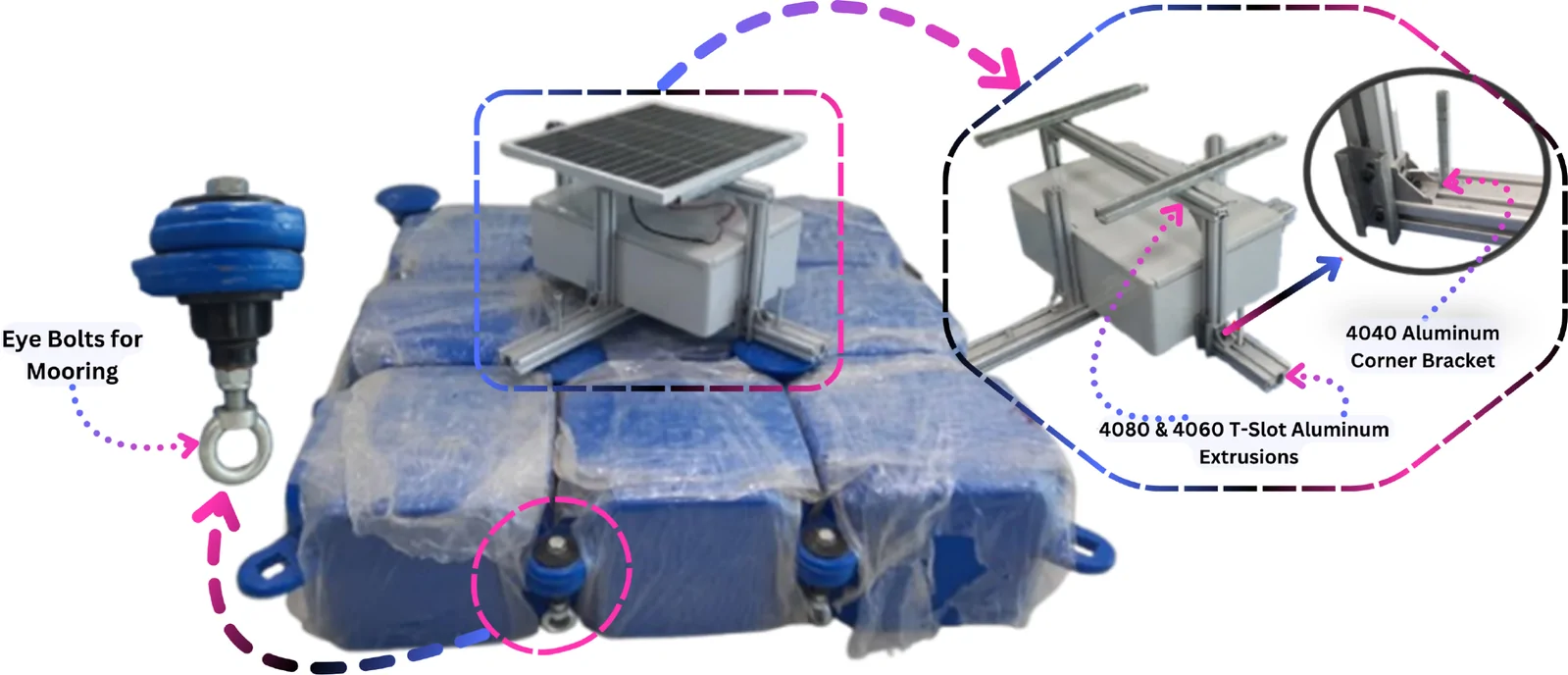
**Structural Assembly of the Floating Sensing Station.** Showing the aluminum framework. The aluminum truss frame, connected via corner brackets. The electronics enclosure and solar power system are elevated on the extrusions.

**Fig. 12:**
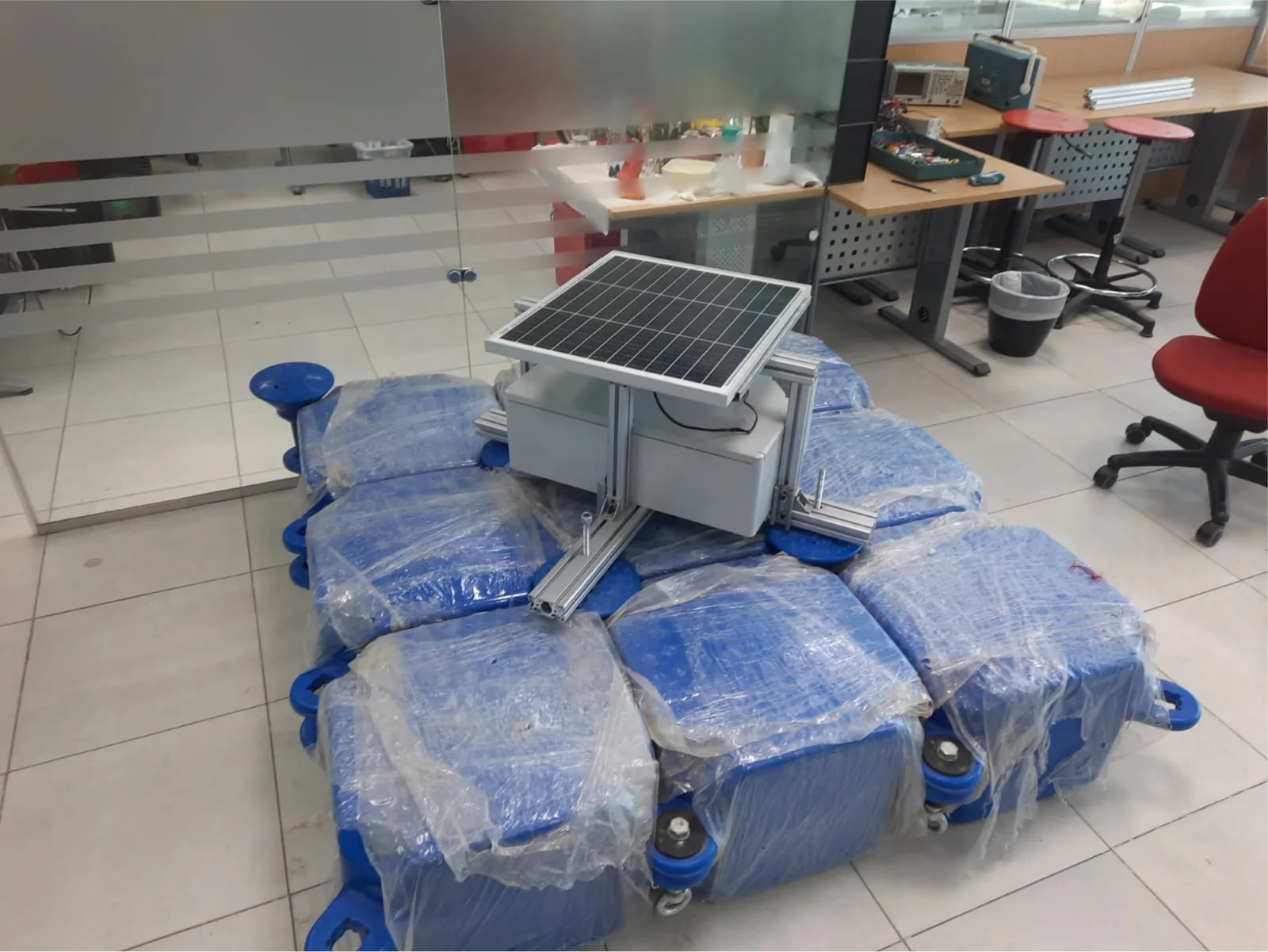
The fully assembled floating hub.

#### 2.4.2 Case A: Single-board autonomous sensing station

Case A uses one floating autonomous sensing station. In this case, the floating platform is an independent, self-powered environmental logging device. One OpenWater Hub board is housed in the IP66 electronics housing in the center of the platform (as shown in Fig. 13).

**Power System:** This autonomous logging device has no connection to the electrical grid; therefore, all of its power comes from energy harvesting.

• **Energy Harvesting:** An 12V photovoltaic panel mounted on the truss connects to an MPPT (maximum power point tracking) commercial solar charge controller.
• **Storage:** The charge controller controls the charging of a 12V 9000mAh lead–acid battery so that there will be enough power available during nighttime or when it is cloudy.
• **Voltage Regulation:** Since the 24V industrial standard for voltage is needed for the RS485 sensor array, the 12V battery output is connected to a DC-DC step-up (boost) converter to regulate the voltage before it reaches the OpenWater Hub power input.

**Operational Logic:** The operating system is designed to use very little power. When the user defines what time interval they want to collect data (i.e., every 15 min), the OpenWater Hub will wake up from its sleep mode. At wake-up time, the OpenWater Hub will activate the RS485 bus, connect to the submerged sensors (pH, temperature, conductivity probes located under the pontoon), read and parse data collected from the submerged sensors, and then send a telemetry packet via the onboard LoRa module back to the shore station. After sending the telemetry packet, the OpenWater Hub will go into low-power sleep mode again.

**Fig. 13:**
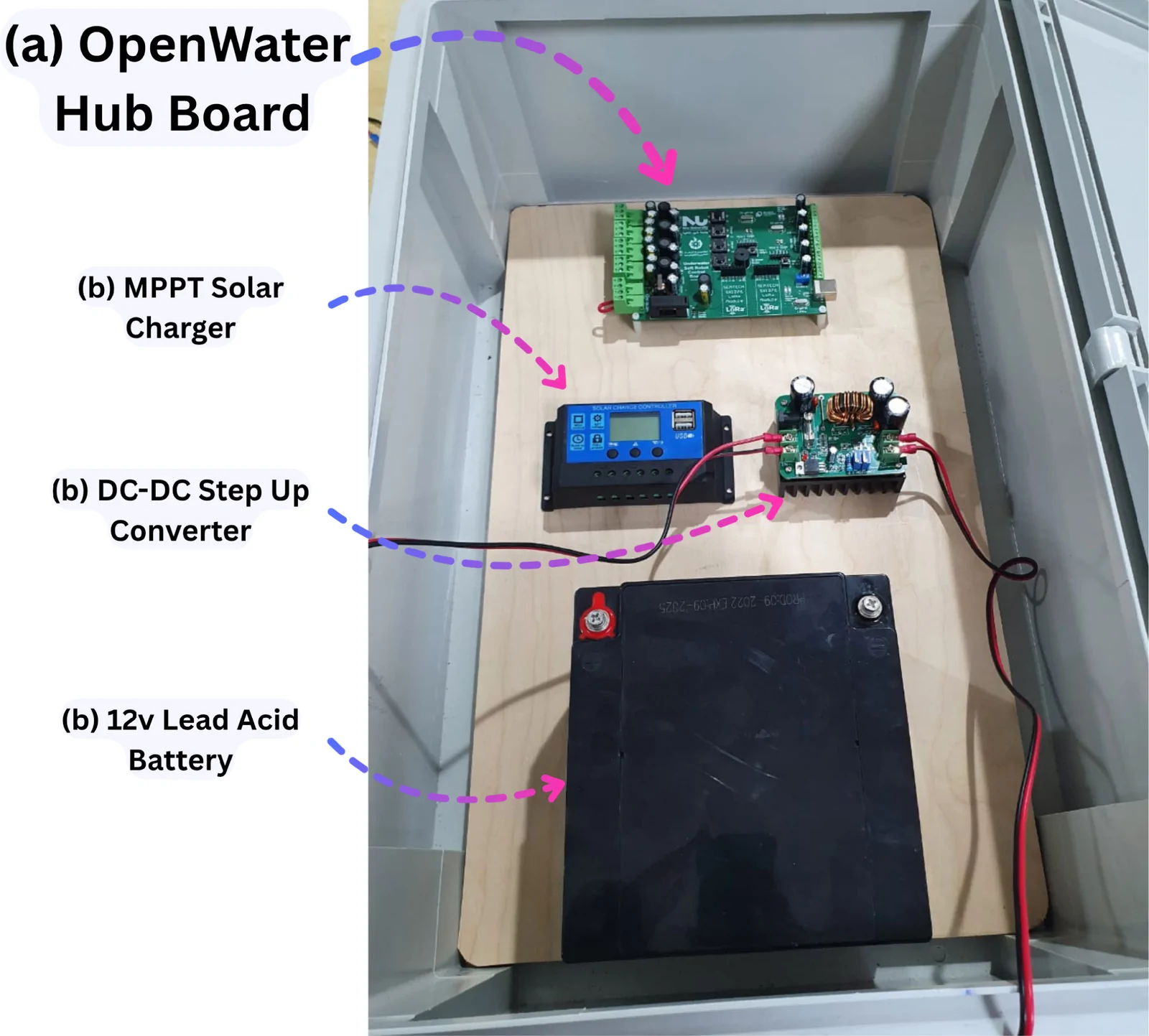
**Internal Architecture of the Floating Sensing Station (Case A).** A top-down view of power and control layout. **(a)** The OpenWater Hub board functions as the central logger and telemetry unit. **(b)** An **MPPT solar charge controller** regulates energy harvesting from the external panel. **(c)** A DC-DC step-up converter boosts the 12V battery output to the 24V required for the sensor bus. **(d)** The 12V lead–acid battery provides the primary energy reservoir for 24/7 operation.

#### 2.4.3 Case B: Dual-board robotic support hub

The Case B configurations demonstrate the scalability of the system. By stacking a second OpenWater Hub board into the enclosure, the station changes to become an active robotic support hub (Fig. 14).

• **Board 1 (Sensing Node):** remains to monitor the environment as in Case A.
• **Board 2 (Robotic Gateway):** supports tethered underwater robotics.
  – **Command Relay:** Board 2 receives commands for movement from the shore via LoRa and forwards those commands down the tether via RS485 to intelligent robots.
  – **Direct Actuation:** For simple, low-cost soft robots lacking onboard intelligence, board 2 can generate PWM signals directly to activate the robot’s actuators via a short tether. As stated in Case B, this method works well for open-loop operation near the sensor but severely attenuates high-frequency PWM signals through long tethers due to capacitive loading and severe signal loss. Therefore, this method is most suitable for short-distance robotic control.
  – **Power Injection:** Battery power supplied to the station is also transmitted down the tether to supply power to underwater robots without onboard batteries. When referencing tether interface cables, power and communication cables used for this application were provided as commercially available standard industrial cables. Each of these cables has a total cross-sectional area of 1.5 ${\text{mm}}^{2}$ per core. Therefore, this should be considered when developing overall system-level power budgets to determine potential voltage drop across the payload under consideration for each robotic application.

**Fig. 14:**
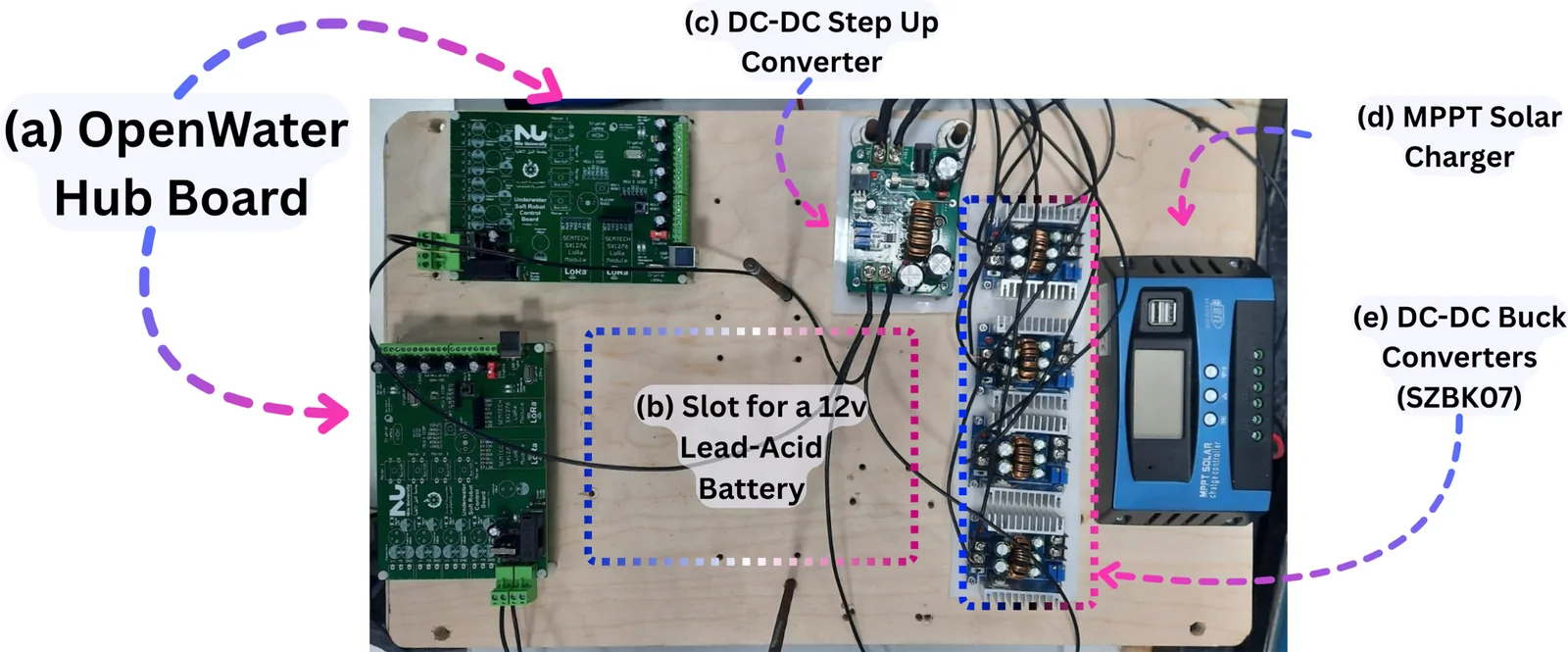
**Case B Dual-Board Architecture.** The expanded configuration for robotic support. **(a)** OpenWater Hub boards, one for sensing operations and one for robotic control logic. **(b)** The slot for the 12V lead–acid battery. **(c)** The DC-DC step-up converter. **(d)** The MPPT solar charger. **(e)** DC-DC buck converters to provide a wide range of power output.

### 2.5 Capabilities summary

The OpenWater Hub eliminates the hardware fragmentation associated with marine research by combining industrial sensor polling and high-current robotics into one printed circuit board. At the electrical level, it provides a dedicated 24 V 24 W power bus to allow for the high inrush currents of mechanical wiper motors. In addition, at the same time, the sensor bus is physically separated from the four 3.0 A adjustable step-down regulators that are controlling the robotic actuators. As well as this separation at the electrical level, the dual ATmega128A architecture ensures strictly timed Modbus RTU operations. Because all serial polling and CRC16 checksum calculations are performed on MCU2, MCU1 continues to operate without interruption and maintain its microsecond-precise kinematics control loops. These two levels of decoupling extend to the telemetry and sensor interfaces. The OpenWater Hub acts as a manufacturer-agnostic control node, which rejects proprietary data logging systems and closed cloud-based infrastructures. Therefore, researchers can send their standardized RS485 data via modular UART socket interfaces using LoRa, Wi-Fi or Bluetooth modules. Researchers will also have complete and unencrypted ownership of high-resolution telemetry data. In summary, these electronics and computer capabilities provide for direct physical scalability. One single open source printed circuit board will be able to scale from a benchtop calibration tool (case zero) to a handheld field lab (case c) to an autonomous offshore environmental logger (case a) and ultimately to an active command relay for tethered underwater robotics (case b). Finally, this unification reduces the amount of capital investment and engineering costs that were historically required to perform multi-domain oceanographic research.

## 3 Design files summary

All open-source design files necessary to replicate the OpenWater Hub, including PCB fabrication Gerbers, mechanical CAD files, and C++ firmware for the various deployment cases, are publicly hosted. Please refer to the OSF repository linked in Table 3 for the complete, version-controlled dataset.

**Table 3:** Summary of open-source design files and firmware.

Design filename	File type	License	Location
**01_Hardware_PCB**	**Directory**	**–**	**OSF.IO Repository Root**
Altium_Source_Files/	Altium Designer PCB layout project folder	CC-BY 4.0	OSF.IO: /01_Hardware_PCB
KiCad_Source_Files/	KiCad PCB layout project folder	CC-BY 4.0	OSF.IO: /01_Hardware_PCB
OpenWaterHub_Schematic_Annotated.pdf	Electronics schematic (PDF)	CC-BY 4.0	OSF.IO: /01_Hardware_PCB
OpenWaterHub_Gerbers.zip	Gerber files for PCB fabrication	CC-BY 4.0	OSF.IO: /01_Hardware_PCB
PCB_Manufacturing_Specs.pdf	Fabrication parameters and thermal limits	CC-BY 4.0	OSF.IO: /01_Hardware_PCB
BOM_M1M2_FullyPopulated.csv	Master Bill of Materials	CC-BY 4.0	OSF.IO: /01_Hardware_PCB
BOM_Actuation_Node_M1_Only.csv	Semi-populated variant BOM (Motors)	CC-BY 4.0	OSF.IO: /01_Hardware_PCB
BOM_Comms_Node_M2_Only.csv	Semi-populated variant BOM (Network)	CC-BY 4.0	OSF.IO: /01_Hardware_PCB
OpenWaterHub_Customization_Limits_Guide.txt	Hardware safety boundaries and limits	CC-BY 4.0	OSF.IO: /01_Hardware_PCB

**02_Mechanical_CAD**	**Directory**	**–**	**OSF.IO Repository Root**
PortableLab_FrontPanel.DXF	2D vector file for acrylic laser cutting	CC-BY 4.0	OSF.IO: /02_Mechanical_CAD
PortableLab_Enclosure_Modifications.x_t	3D Parasolid CAD for chassis modification	CC-BY 4.0	OSF.IØ: /02_Mechanical_CAD

**03_Firmware**	**Directory**	**–**	**OSF.IO Repository Root**
OpenWaterHub_Sensor_Test.ino	Base RS485 Modbus validation sketch	CC-BY 4.0	OSF.IO: /03_Firmware
MCU1_PermenantSleep.ino	Actuation node state machine	CC-BY 4.0	OSF.IO: /03_Firmware
MCU2_PermenantSleep.ino	Communications node baseline sleep logic	CC-BY 4.0	OSF.IO: /03_Firmware
MCU2_Dynamic_PwrSave_Packets.ino	Sensor node polling and deep-sleep routine (Cases A & C)	CC-BY 4.0	OSF.IO: /03_Firmware
MCU2_PontoonSerialRelay.ino	RS485 tethered bridge logic (Case B)	CC-BY 4.0	OSF.IO: /03_Firmware
esp32_portableStationCode.ino	ESP32 cloud connectivity and telemetry	CC-BY 4.0	OSF.IO: /03_Firmware

**04_HMI_Display**	**Directory**	**–**	**OSF.IO Repository Root**
DWIN_DGUS_Project.rar	Touch screen interface source files	CC-BY 4.0	OSF.IO: /04_HMI_Display

## 4 Bill of materials summary

The component specifications and hardware costs are categorized across six tables to reflect the modular deployment options. These include the fully-populated core PCB (Table 4), semi-populated actuation and communication boards (Table 5, Table 6), the portable field laboratory (Table 7), the autonomous floating platform (Table 8), and optional sensor peripherals (Table 9).

**Table 4:** **OpenWater Hub PCB Electronics (Fully-Populated M1+M2 Architecture).** Costs are approximate based on global distributors (e.g., DigiKey, LCSC) at the time of publication.

Designator	Component	No.	Unit Cost (USD)	Total (USD)	Source	Material type
M1, M2	ATmega128A-AU 8-bit Microcontroller	2	$4.51	$9.02	LCSC	Semiconductor

U1	MAX487-TD RS485 Transceiver	1	$0.25	$0.25	LCSC	Semiconductor

M3	CH340C USB-to-UART Bridge (Integrated 12 MHz)	1	$0.50	$0.50	LCSC	Semiconductor

VR2	LM2576-5.0 Fixed Voltage Regulator (5 V)	1	$2.35	$2.35	LCSC	Semiconductor

VR1, VR3, VR4, VR5	LM2576-ADJ Adjustable Voltage Regulator	4	$0.35	$1.40	LCSC	Semiconductor

D12	PSM712 Asymmetric TVS Array (RS485 ESD Protection)	1	$0.04	$0.04	LCSC	Semiconductor

D7	SMAJ24A Transient Voltage Suppressor (24 V)	1	$0.05	$0.05	LCSC	Semiconductor

T1	UU9.8 Common-Mode Choke	1	$0.31	$0.31	LCSC	Ferrite/Copper

L1–L5	100μH Power Inductors	5	$0.10	$0.50	LCSC	Ferrite/Copper

C8, C9, C15…	1000μF/16 V Electrolytic Bulk Capacitors	9	$0.14	$1.26	LCSC	Aluminum Electrolytic

Various (Full-Sized BOMs are provided as CSV files with paper)	Commodity Passives (Resistors, MLCCs, Diodes, Headers)	∼150	N/A	$5.89	LCSC	Thick Film/Ceramic

**BOM Subtotal**	**Raw Component Material Cost**	–	–	**$21.57**	–	–

PCB & SMT	Bare Board (TG150/ENIG) & Turnkey Assembly Labor	1	$24.03	$24.03	PCBWay	Composite/Labor

**TOTAL**	**Fully Assembled PCB Total**	–	–	**$45.60**	–	–

**Table 5:** **OpenWater Hub PCB Electronics (Semi-Populated with Actuation Node M1 Only).** Costs are approximate based on global distributors (e.g., DigiKey, LCSC) at the time of publication.

Designator	Component	No.	Unit Cost (USD)	Total (USD)	Source	Material type
M1	ATmega128A-AU 8-bit Microcontroller	1	$4.51	$4.51	LCSC	Semiconductor

VR2	LM2576-5.0 Fixed Voltage Regulator (5 V)	1	$2.35	$2.35	LCSC	Semiconductor

VR1, VR3, VR4, VR5	LM2576-ADJ Adjustable Voltage Regulator	4	$0.35	$1.40	LCSC	Semiconductor

D7	SMAJ24A Transient Voltage Suppressor (24 V)	1	$0.05	$0.05	LCSC	Semiconductor

T1	UU9.8 Common-Mode Choke	1	$0.31	$0.31	LCSC	Ferrite/Copper

L1–L5	100μH Power Inductors	5	$0.10	$0.50	LCSC	Ferrite/Copper

C8, C9, C24…	1000μF/16 V Electrolytic Bulk Capacitors	8	$0.14	$1.09	LCSC	Aluminum Electrolytic

Various	Various (Full-Sized BOMs are provided as CSV files with paper)	∼80	N/A	$3.94	LCSC	Thick Film/Ceramic

**BOM Subtotal**	**Raw Component Material Cost**	–	–	**$14.15**	–	–

PCB & SMT	Bare Board (TG150/ENIG) & Turnkey Assembly Labor	1	$24.03	$24.03	PCBWay	Composite/Labor

**TOTAL**	**Semi-Populated PCB Total**	–	–	**$38.18**	–	–

**Table 6:** **OpenWater Hub PCB Electronics (Semi-Populated with RS485 Node M2 Only).** Costs are approximate based on global distributors (e.g., DigiKey, LCSC) at the time of publication.

Designator	Component	No.	Unit Cost (USD)	Total (USD)	Source	Material type
M2	ATmega128A-AU 8-bit Microcontroller	1	$4.51	$4.51	LCSC	Semiconductor

U1	MAX487-TD RS485 Transceiver	1	$0.25	$0.25	LCSC	Semiconductor

M3	CH340C USB-to-UART Bridge (Integrated 12 MHz)	1	$0.50	$0.50	LCSC	Semiconductor

VR2	LM2576-5.0 Fixed Voltage Regulator (5 V)	1	$2.35	$2.35	LCSC	Semiconductor

D12	PSM712 Asymmetric TVS Array (RS485 ESD Protection)	1	$0.04	$0.04	LCSC	Semiconductor

D7	SMAJ24A Transient Voltage Suppressor (24 V)	1	$0.05	$0.05	LCSC	Semiconductor

T1	UU9.8 Common-Mode Choke	1	$0.31	$0.31	LCSC	Ferrite/Copper

L2	100μH Power Inductor	1	$0.10	$0.10	LCSC	Ferrite/Copper

Various	Various (Full-Sized BOMs are provided as CSV files with paper)	∼50	N/A	$2.61	LCSC	Thick Film/Ceramic

**BOM Subtotal**	**Raw Component Material Cost**	–	–	**$10.72**	–	–

PCB & SMT	Bare Board (TG150/ENIG) & Turnkey Assembly Labor	1	$24.03	$24.03	PCBWay	Composite/Labor

**TOTAL**	**Semi-Populated PCB Total**	–	–	**$34.75**	–	–

**Table 7:** **Case C - Portable “Lab-in-a-Box” Assembly.** Details the components required to convert the core PCB into a portable field unit.

Designator	Component	No.	Unit Cost (USD)	Total (USD)	Source	Material type
PCB-01	OpenWater Hub PCB (From Table 4)	1	$45.60	$45.60	Self-Built	Electronics

MECH-01	Aluminum Tool Case (Equivalent to EGA Master 51012, 455 × 205 × 320 mm)	1	$50.00	$50.00	Local/Generic	Aluminum/Composite

MECH-02	Custom Laser-Cut Front Panel	1	$15.00	$15.00	Local Fab Lab	Acrylic (PMMA)

HMI-01	DWIN HMI Touch Display (7-inch)	1	$45.00	$45.00	DWIN/Aliexpress	Electronics

PWR-01	12 V 9 Ah Sealed Lead-Acid Battery	1	$22.00	$22.00	Local Supplier	Chemical

PWR-02	PWM Solar Charge Controller (10 A)	1	$18.00	$18.00	Generic	Electronics

PWR-03	150 W DC–DC Boost Converter (12 V to 24 V)	1	$8.00	$8.00	Generic	Electronics

PWR-04	24 V Industrial DC Power Supply (AC Input)	1	$25.00	$25.00	Mean Well	Electronics

SAFE-01	2 A DC Circuit Breakers	3	$3.00	$9.00	Generic	Electromechanical

SW-01	3-Position Rotary Selector Switch	1	$5.00	$5.00	LCSC	Plastic/Metal

CON-01	GX16 4-Pin Aviation Connectors (Splashproof)	16	$1.50	$24.00	Generic	Metal

CON-02	IEC C14 AC Power Socket & Circular DC Jack	1	$3.00	$3.00	Generic	Metal/Plastic

PNL-01	Panel-Mount USB-B Extension, LEDs, Pushbuttons	1	$8.00	$8.00	Generic	Electronics/Metal

MISC-01	Misc. Hardware (18 AWG Wire, M3 Standoffs, Terminals)	1	$15.00	$15.00	Local Supplier	Copper/Metal

**TOTAL**	**Portable Lab Assembly Total**	–	–	**$292.60**	–	–

**Table 8:** **Cases A & B - Floating Sensing Station Assembly.** Details the total materials and cost required to build the autonomous offshore platform.

Designator	Component	No.	Unit Cost (USD)	Total (USD)	Source	Material type
PCB-01	OpenWater Hub PCB (From Table 4)	1	$45.60	$45.60	Self-Built	Electronics

PONT-01	HDPE Modular Pontoon Cubes	9	$28.00	$252.00	Marine	HDPE

TRUSS-01	4040/4080 Aluminum T-Slot Extrusion	∼6m	$8/m	$48.00	Local	Aluminum

BOX-01	IP66 Waterproof Electronics Enclosure	1	$35.00	$35.00	Local	ABS

SOL-01	50 W 12 V Monocrystalline Solar Panel	1	$55.00	$55.00	Local	Silicon

PWR-01	12 V 9Ah Sealed Lead-Acid Battery	1	$22.00	$22.00	Local	Chemical

PWR-02	MPPT Solar Charge Controller	1	$18.00	$18.00	Amazon	Electronics

**TOTAL**	**Floating Station System Total**	–	–	**$475.60**	–	–

*Note: For Case B (Robotic Support Hub), a second OpenWater Hub PCB is added, increasing the total by $45.60.

**Table 9:** **Optional Peripherals (Sensors & Wireless).** The system is compatible with any RS485 sensor; the items listed here are validated optional payloads.

Designator	Component	No.	Unit Cost (USD)	Total (USD)	Source	Material type
SEN-01	JXCT Water EC Sensor (RS485)	1	$90.00	$90.00	JXCT IoT	Industrial Probe

SEN-02	JXCT Water pH Sensor (RS485)	1	$110.00	$110.00	JXCT IoT	Industrial Probe

SEN-03	JXCT Dissolved Oxygen Sensor (RS485)	1	$140.00	$140.00	JXCT IoT	Industrial Probe

COM-01	DRF1278DX LoRa Radio Module (433MHz)	1	$12.00	$12.00	Dorji/eBay	Electronics

COM-02	HC-05 Bluetooth Serial Module	1	$4.00	$4.00	Generic/eBay	Electronics

ANT-01	433MHz SMA Antenna (3dBi)	1	$5.00	$5.00	Generic	Plastic/Metal

**TOTAL**	**Peripherals Total**	–	–	**$357.00**	–	–

## 5 Build instructions

The **OpenWater Hub system** has been developed under the guidance of three separate stages: (i) assembly of the printed circuit board (PCB); (ii) integration of the portable Lab-in-a-Box; and (iii) construction of the floating sensing station. All electronic components described in the following paragraphs relate to the component designators presented in the **Bill of Materials** (Section 4) and the PCB schematic.

### 5.1 Phase 1. OpenWater hub PCB assembly.

**Caution**: Use **surface-mount technology (SMT)**. Always provide proper ventilation during the soldering operation and use **ESD-safe tools** to avoid damaging the microcontrollers.

1. **Fabrication of PCB:** Send the bare PCB to a fabrication house of your choice, using the Gerber files included in the repository (OpenWaterHub_Gerber.zip). The designed PCB is a **standard FR4 2-layer design** (thickness = 1.6mm, copper = 1oz) which can be easily fabricated by low-cost fabrication houses. In this context, we refer to the **bare PCB** as only the manufactured fiberglass board before any electronic components are attached by means of soldering.
2. **Strategy for Component Population and Variants:** Component population may vary depending on how you intend to deploy the board. In order to boot the board, researchers must populate the core 24 V input filter, 5 V logic regulator, and UVLO components.
  • **Fully Populated**: By populating all components, researchers will obtain a **complete sandbox board** that can drive motors and parse RS485 data at the same time (Case Zero).
  • **Variant A (Standalone Actuator)**: Researchers who want to create a standalone motor driver can semi-populate the board by soldering only the actuation MCU, motor regulators, and bulk capacitors and omitting RS485 ICs entirely.
  • **Variant B (RS485 Sensor Node)**: Researchers who wish to create a standalone RS485 gateway can semi-populate the board by soldering only the RS485 sensor MCU, MAX487 transceiver, and USB bridge and leave the entire motor-driving circuitry empty.
3. **SMD Soldering:** Depending on your selected build variant, start by soldering the necessary SMD components.
  • Align and solder the two **ATmega128A microcontrollers** (M1,M2). Verify that pin 1 on each chip is oriented correctly.
  • Solder the **MAX487 transceiver** (U1) and **CH340G USB bridge** (M3)
  • Solder the smaller SMD passive components (capacitors, resistors, and LEDs) identified in the assembly drawing (OpenWaterHub_Assembly.pdf) .
4. **Through-Hole Soldering:** Once all SMD components have been securely fastened, continue with through-hole components.
  • Install the **voltage regulators** (VR1-VR5). These vertical units need to be carefully aligned so they do not span across adjacent pads.
  • Install the **screw terminals** (J3,J7,J10,J11) used for motor connections and the primary **RS485 block**.
  • Install the **USB Type-B connector** (J12) and **wireless module headers** (H3,H4).
5. **Cleaning and Verification:**
  • Clean the board with **isopropyl alcohol** to remove flux residues after soldering.
  • Verify there are no **shorts** between VCC and GND planes using a multimeter before applying power.
  • Connect a 24V-V supply to the input terminal. Check that 5V-V logic plane is stable.

### 5.2 Phase 2. Assembly of portable “Lab-in-a-Box” (Case C)

1. **Preparation of Enclosure:**
  • Remove all tool dividers or foam inserts from inside the aluminum tool box (equivalent to EGA Master 51012, 455×205×320mm) (MECH-01)
  • Laser-cut a custom acrylic front panel (MECH-02).
  • Fabricate an internal mounting tray (using 3D-printed brackets or aluminum rails) to attach the battery and electronics to the underside of the case using M3 spacers and hardware (MISC-01).
2. **Integration of Front Panel Components:**
  • Mount the DWIN touch screen (HMI-01) to backside of acrylic panel using M3 screws.
  • Install 16× GX16 aviation connectors (CON-01) into their respective holes located on the right side of the panel.
  • Attach rotary selector switch (SW-01), IEC C14 AC power socket, and circular solar DC input jack (CON-02).
  • Install panel-mount USB-B extension, status LEDs, and push buttons (PNL-01) into their assigned acrylic cut-outs.
3. **Wiring of Power Systems:** Follow the wiring diagram in the design repository for actual pin assignments.
  • **AC Path:** Connect AC socket to input side of 24 V industrial PSU (PWR-04). Connect PSU output to “grid” terminal of selector switch (SW-01), inserting a 2 A DC circuit breaker (SAFE-01) in series for overcurrent protection.
  • **Solar Path:** Connect solar DC input to MPPT charge controller (PWR-02). Connect lead–acid battery (PWR-01) to controller’s battery terminals.
  • **Boost Stage:** Connect “Load” output of charger controller to input side of DC–DC boost converter (PWR-03). Adjust converter output voltage level to exactly 24 V. Connect this output to the “solar” terminal of the selector switch, attaching a second 2 A DC circuit breaker (SAFE-01).
  • **Main Output:** Connect the common output of the selector switch to the power input terminals of the OpenWater Hub PCB using 18 AWG wire and crimp terminals (MISC-01).
4. **Cable Routing:**
  • Connect wires from the rear pins of 16GX16 connectors to a single RS485 bus (A, B, VCC, GND). Connect this bus to RS485 terminal on PCB.
  • Connect DWIN screen’s data cable to the dedicated RS485 interface port (J1) on center PCB.
  • Connect panel-mounted USB-B extension, LEDs, and reset switches (PNL-01) to respective PCB headers.
  • Secure panel to case frame using self-tapping screws.
  For details regarding actual electrical connectivity between external user interfaces and PCB components, please refer to Fig. 15.

**Fig. 15:**
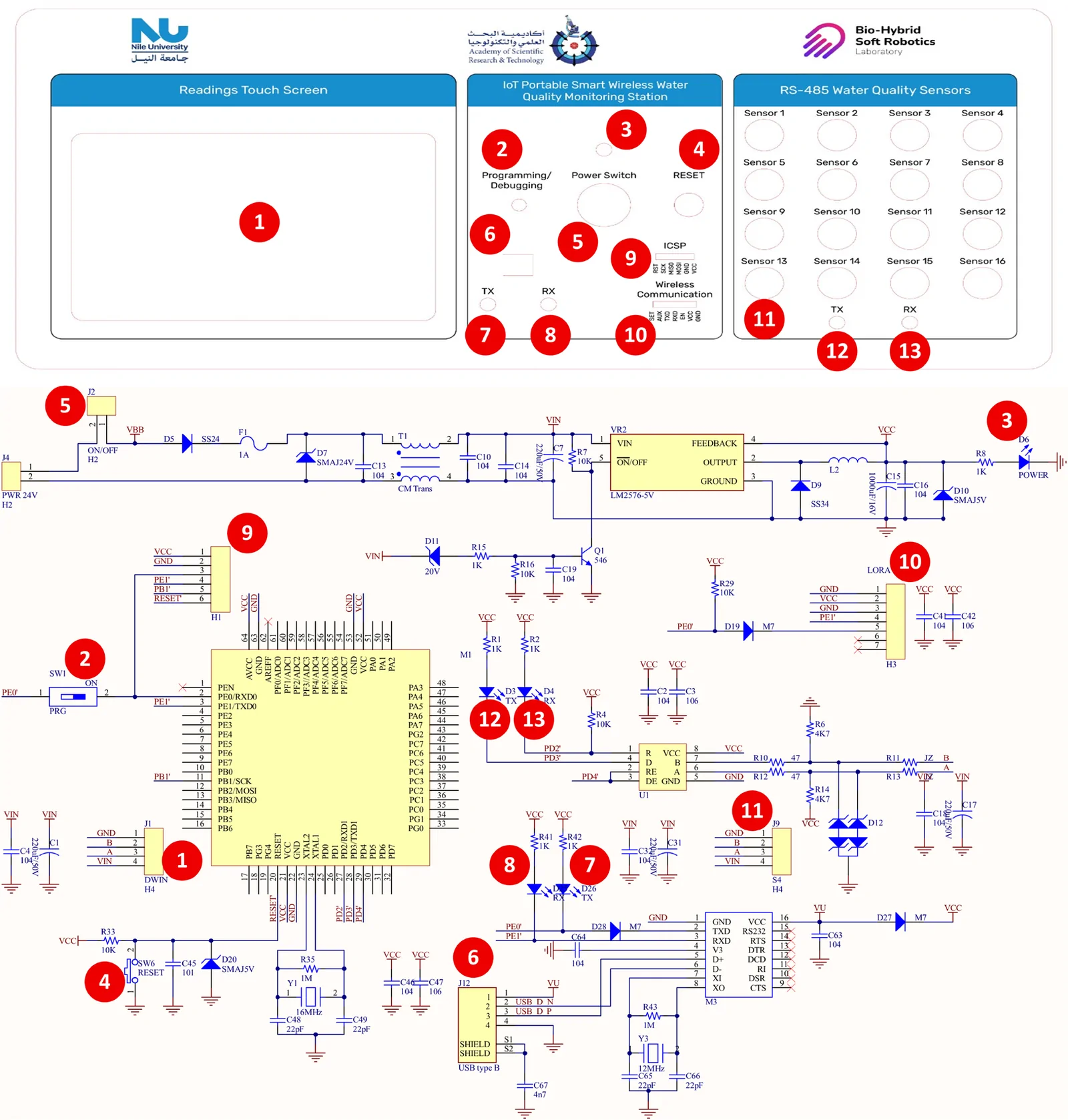
**Portable Lab-in-a-Box Front Panel Electrical Connection Diagram.** This figure illustrates the mapping of physical electrical connections between user interface components externally installed onto the acrylic front panel and internally connected components on the PCB. Primary connections are: (1) DWIN touch screen via UART (H4); (2) programmer enable switch; (3) power LED; (4) system reset button; (5) primary power switch; (6) USB-B data port; (7–8) TX/RX Status LEDs for serial data bus; (9) ICSP header; (10) LoRa wireless module socket; and (11–13) RS485 sensor bus with associated status LEDs.

### 5.3 Phase 3. Assemble floating station (Cases A & B)

1. **Construction of Pontoon Platform:** Arrange nine HDPE pontoon cubes (PONT-01) together to form an approximately 1.5×1.5m platform. Insert connection pins into locking lugs. **Crucial Note:** At all junction points where the structural frame will connect to pontoon cubes, insert standard **anchor pins** (see Fig. 10) instead of regular pins. Standard anchor pins have a stainless steel threaded rod embedded within them for securing fixation of the aluminum truss structure. Lock all pins together using standard pontoon keys, resulting in a rigid floating platform.
2. **Assembling Truss Frame Structure:** Cut sections of 4080-profile aluminum extrusions (TRUSS-01) to produce base square diagonals matching the dimensions of 4 anchor point centers. Mount the base frame structure to the threads of the anchor pins. Construct columns using 0.35 m long sections of 4040-profile extruded aluminum and corner bracket assembly forming elevated support for electronics compartment and solar panel.
3. **Installation of Electronic Components:** Mount IP66 enclosure (BOX-01) vertically along aluminum column members. Install solar panels (SOL-01) on top of the truss at an angle 45° (optimal angle depends on the latitude of the deployment site). Inside the enclosure, mount the OpenWater Hub PCB, MPPT controller, and battery. Drill cable gland holes in the bottom plate of the enclosure for sensor cables and robot tether. Ensure all glands are tightened sufficiently to meet IP67 ratings. Connect solar panels to MPPT controllers and batteries to systems per identical logic as in portable Lab, excluding AC mains paths. Sealing of cable penetrations for sensor arrays and robot tethers are performed using PG9 rated IP67 cable glands, maintaining watertight integrity of the enclosure.

## 6 Operation instructions

The OpenWater Hub is an open-hardware design that can be programmed using the Arduino IDE. Below are the one-time procedures for installing the bootloader, development environment, and an example firmware sketch illustrating how to poll sensors on the platform.

### 6.1 One-time bootloader and fuse configuration

Firstly, before the OpenWater Hub can be programmed using the Arduino IDE, both ATmega128A processors need to be loaded with bootloaders and their appropriate fuses. This is a one-time process.

1. **Hardware Connections**: Connect a **USBasp programmer** (or other AVR programmer) to the **6-pin ICSP header** for MCU1 on the OpenWater Hub PCB. All six pins are arranged according to the standard AVR pinout: MISO, MOSI, SCK, RST, VCC, and GND.
2. **Tool Chain**: Confirm you have **AVRDUDE** installed and accessible via your system’s command-line tool. AVRDUDE is the utility used to interact with the programmer.
3. **Bootloader Firmware**: The firmware is designed to operate with the **MegaCore bootloader** by MCUdude. MegaCore provides a method for serial programming that has been optimized for the ATmega128 series.
4. **Fuse Settings**: Using AVRDUDE, set the fuses for **ATmega128A**. The settings above determine how the microcontroller uses an external crystal for timing and enables serial programming. Run these parameters:
  • **Low Fuse Byte:** 0 × 9F
  • **High Fuse Byte:** 0xCE
5. **Burn Bootloader**: Once the fuses have been set, burn the **pre-compiled bootloader .hex file** into the processor either directly using AVRDUDE or by using the **Arduino IDE burn bootloader option** under the *Tools* menu.
6. **Repeat for MCU2**: Repeat steps 1–5 for **MCU2** using its dedicated ICSP header.

### 6.2 The development environment

Before the OpenWater Hub can be programmed using the Arduino IDE, each ATmega128A microcontroller needs to be flashed with a bootloader and configured with appropriate fuse settings. This is a one-time procedure.

1. **Install IDE**: Download and install the **Arduino IDE** (version 1.8.x or later).
2. **Add New Board Support**: Launch the Arduino IDE and select *File* > *Preferences*. Add the **MegaCore URL** to the “Additional Boards Manager URLs” input box. Next, browse to *Tools* > *Board* > *Boards Manager*. Search for **MegaCore**, then click *Install*.
3. **Configure IDE Settings to Match HW Specifications**: Configure the IDE settings to correspond to the OpenWater Hub hardware configuration:
  • **Board:** MegaCore > ATmega128
  • **Clock:** 16MHz external clock
  • **BOD:** 2.7V
  • **LTO Compiler Option:** Enabled
  • **Bootloader:** Yes (UART0)
4. **Upload Firmware**: After performing the initial bootloader upload via ICSP, subsequent firmware uploads will be uploaded directly to the **USB Type-B port**.
  • Connect the board to a PC using a typical USB cable.
  • Select the corresponding COM port under *Tools*.
  • Press *Upload*. An auto-reset circuit on-board will activate the bootloader and upload the firmware. To avoid data collisions between the CH340 USB bridge and external UART devices or FTDI programmers connected to header H1, users must physically toggle the SW1 DIP switch to sever the RX/TX traces, ensuring clean code uploads to MCU2.
  *Note: The onboard USB Type-B port is hardwired exclusively to MCU2 via the CH340 bridge. Subsequent firmware updates for the RS485 sensor node (MCU2) can be flashed directly over this USB connection. Because the microcontrollers are physically isolated, firmware updates for the actuation node (MCU1) must always be flashed using an external serial programmer connected to header H2.*

### 6.3 Profiles for deployments of firmware

In transitioning from lab validation to autonomous offshore deployments of the OpenWater Hub, there are multiple modular sets of C++ firmware profiles available. Unlike traditional single codebase designs, because of the two processors available on this design, developers will create separate logic states for MCU1, MCU2, and the communication modules depending on which of three possible physical cases they are in.

#### 6.3.1 Verification of initial system performance

During initial system validation, developers should employ OpenWaterHub_Sensor_Test.ino. This base test sketch verifies the physical RS485 layer and logic states of MAX487 transceivers by continuously sending Modbus RTU remote terminal unit requests over four connected probes and validating local CRC16 checksums generated by each probe. Each outputted raw hex byte string is displayed in hexadecimal format in real-time in the serial monitor to allow users to identify any potential issues with wiring prior to going out into field testing.

#### 6.3.2 Environmental sampling and long-term sleep

As part of the autonomous floating station (Case A) and portable laboratory (Case C), MCU2_Dynamic_PwrSave_Packets.ino represents operational logic for sampling environments and long-term sleeping. Based on a defined interval sampled from an internal watchdog timer (WDT), this firmware determines when samples are taken. Due to a restriction in physical implementation where sample cycles cannot exceed 2.1 s due to the WDT limitations of an ATmega128A processor, a unique memory preservation strategy was employed. The code specifies a sleep-cycle counter variable that resides in memory space preserved from initialization using .noinit compiler directives. The variable is protected from being initialized to zero by C++’s start-up routine during WDT hardware resets. Thus, an additional memory area is reserved that preserves sleep duration counters without affecting EEPROM reserves. Following each sample cycle, MCU2 creates a comma-separated string containing all of the collected data from the sensors, sends it out via hardware UART, and enters a short listen period (approximately 1.5 s) that waits for any pending top-side commands sent back via dashboards before entering another long-term sleep cycle.

#### 6.3.3 IoT integration and data transfer

Data transfer from the sensor array is handled by esp32_portableStationCode.ino operating on the secondary wireless module. It reads in each packet received by serial input from MCU2, identifies sensor readings embedded within packets, and performs MQTT publish to send each reading to an Arduino IoT cloud.

#### 6.3.4 Persistent network connectivity

The ESP32 maintains persistent network connectivity; additionally, it periodically sends pending reboot signals from MCU2 down the UART wire during periods of active listening.

#### 6.3.5 Command relay between surface and underwater robotics

If deployed as a robotic support hub (Case B), MCU2 operates MCU2_PontoonSerialRelay.ino. This firmware configures MCU2 as a bi-directional, non-blocking half-duplex relay. Rapidly alternating driver enable pins of the transceivers allow MCU2 to capture BT/RF commands from an operator at surface level and immediately forward them down 50-meter RS485 tether to an underwater robot actively moving underneath.

#### 6.3.6 Locking down power consumption and pin anchoring

To minimize unwanted current drain caused by unprogrammed microcontrollers in a power-down state, users may load MCU1_PermanentSleep.ino and MCU2_PermanentSleep.ino into MCU1 and MCU2 respectively. These strict shutdown scripts turn off ADC peripherals on MCUs and stop hardware timers. They anchor some GPIOs as high-impedance (High-Z) barriers against onboard LEDs and external pull-up resistors to ensure that MCUs enter a permanently power-down state.

### 6.4 Operation safety requirements

Users must strictly comply with several electrical requirements during field deployments and hardware configurations:

• **Actuator Power Sourcing:** Users must never activate PWM outputs for actuation while powering the board exclusively from the USB-B port. Users must first make sure that an external 24 V supply source exists before activating PWM outputs.
• **Termination Resistance on RS485 Bus:** If a user deploys cables longer than 10 m for underwater communication, users must add a 120 Ω terminating resistance directly onto the end node of each sensor channel in order to suppress signal reflections and prevent frame corruption from Modbus RTU.
• **Power Gating Sensors:** Users must apply high-side switching (cut-off supply voltage) for power savings on each sensor plane via a P-Channel MOSFET. Applying low-side N-Channel MOSFET switching cuts off ground references. Immediately after applying power gating MOSFET switching users will destroy MAX487 transceivers due to damage resulting from immediate destruction of return currents from 24 V supplies traveling backward through RS485 data lines.

## 7 Validation and characterization

### 7.1 Power profile and autonomy

A broad systems-level power budget was conducted to determine how much energy will be consumed, provide a means to define the component-level static footprint of each element of the system and develop mathematical models for calculating the ability of the system to operate autonomously using no external power source. In order to measure the milliwatt-level (mW) current draw of each element of the system, we utilized high-resolution benchtop profiling using a HAMEG HMP2030 programmable power supply. We also developed an empirical method to evaluate two different power configurations: a 22 V direct input simulating shore power (Case C) and a 12 V boosted input (stepped up to 22 V) simulating the offshore marine battery architecture (Cases A and B). With respect to operation at 22 V, there are benefits to this choice of operating voltage. It is situated well within the board’s narrow 20 V – 24 V safe operating window, which leaves room for some voltage drop along the tethers connecting the sensors to the board while still providing enough headroom so that when the transients occur (e.g., switching spikes, etc.), they do not exceed the 24 V transient voltage suppression threshold.

Static component profiling provides a method to measure the baseline control logic of the RS485 Sensor Node at 196.4 mW (Fig. 16). The RS485 sensor node is representative of the core communication structure of the platform. Evaluation of a completely populated board measures the physical impact that expansion of this core node has upon completing a system. Leaving both the actuation MCU and the unused PWM motor driver(s) on the PCB but allowing them to operate in a pure data logging mode, adds an additional idle drain of 742 mW to the overall system power profile. When we turn on the DC-DC boost converter to convert the 12 V battery output into a 22 V bus, it results in a thermal conversion penalty of 196.9 mW. Telemetry overhead establishes an upper bound for the power budget while continuing to maintain a continuous Wi-Fi connection through the use of the ESP32 module and communicating with the Arduino IoT Cloud, requires a continuous 537.9 mW. Replacing the ESP32 with the AS32-TTL-100 LoRa module reduces telemetry footprint to 125 mW. The industrial sensors have fixed static draws: temperature (111.4 mW), dissolved oxygen (196.5 mW), and pH (270 mW).

Dynamic power profiling relates these static values to a high-resolution 30-second polling cycle (Fig. 17). The test plot includes four different configurations: An ESP32 node with three sensors, a LoRa node with three sensors, a baseline node with three sensors (no telemetry), and the RS485 sensor node itself – without any other elements or sensors attached – as a basic operational reference point. Each configuration uses the watchdog timer (WDT) to govern the state machine. The ESP32 configuration has a very high continuous baseline (∼1500 mW) due to continued IoT cloud connection. Advanced deep-sleep routines were purposefully removed from the ESP32 firmware in order to prioritize network stability during testing. On the other hand, the LoRa configuration goes down to a nearly baseline low-point (∼1100 mW). Although an ultra-deep-sleep firmware was not installed into the LoRa module itself, putting the main MCU into deep-sleep stops all serial transmission activity, thus removing most of the active transmission overhead of the LoRa module, except for those periodic polling bursts where transmission occurs.

The mathematical extrapolations of these profiles define what constitutes “dark endurance”, i.e., what is the longest period of time that the system can continue to operate with no solar input (Fig. 18). The dark endurance was determined by relating the previously measured current loads against a standard 600-second (or 10-minute) polling interval. As stated before, the physical system contains a single 12 V, 9 Ah sealed lead–acid (SLA) battery with a theoretical maximum capacity of 108 Watt-Hours (Wh).

The duty cycle consists of three periods: polling (5 s), transmission (2 s), and sleep (593 s). By converting the measured milliwatt levels to joules (J), one may isolate the energy per cycle. For example, with the ESP32+3 sensors configuration:

Polling:

(2) 1.6641W×5s=8.320J

Transmission:

(3) 2.2656W×2s=4.531J

Sleep:

(4) 1.4591W×593s=865.250J

Eqs. (2), (3), (4), make it clear that the sleep period dictates the operating boundaries of the power budget. Given continuous power to each element, during each 593-second sleep period, each electrochemical probe introduces a combined 577.9 mW load (pH: 270.0 mW, DO: 196.5 mW, Temp.: 111.4 mW). The theoretical autonomy of a LoRa configuration with three sensors attached is limited to approximately 4.12 days when operated on a battery having a capacity of 108 Wh. However, there exists several options available to increase this endurance using modifications outlined in Section 2.1.4. One such option involves physically decoupling each of the sensors from the high-voltage side of the 24 V rail using the PB1-controlled MOSFET, thereby removing their static drain from the battery during each sleep period. By doing so, each sensor will only consume high currents for each short-duration polling (i.e., ∼7 s) and save ∼13.7 Wh/day in total energy consumption. Reducing total system consumption from 26.22 Wh/day to 12.51 Wh/day increases calculated endurances from 8.63 days to approximately 8.83 days, respectively, and drives the fully populated sensor array closer to its theoretical maximum endurance of ∼8.83 days when operated without any sensors.

In addition, similar proportional extensions apply to ESP32 configurations.

Realistic field endurance can only be realized if depth-of-discharge (DoD) limitations are placed on each of the chemical battery pools. For example, typical sealed lead–acid (SLA) chemistry (50% DoD limit) reduces usable energy to 54 Wh, reducing calculated endurance times to 1.53 days (ESP32) and 2.06 days (LoRa), respectively (half the operational time).

Finally, upgrading to lithium-ion chemistry (80% DoD limit) yields usable energy of 86.4 Wh, increasing SLA limits by greater than 60% within the same physical footprint.

Integration with solar panels converts these finite boundaries into theoretically infinite autonomous operation. Daily solar yield is calculated mathematically as follows:

(5) Yield (Wh/day)=Panel Rating (W)×Peak Sun Hours (h)×System Efficiency

Given a marine-based baseline of 4 peak sun hours and applying a conservative efficiency multiplier of 70% to account for MPPT and chemical conversion losses, a minimum rated panel of 10 W yields a daily solar yield of 28 Wh/day. This amount is sufficient to satisfy the required power needs of the LoRa configuration but falls short of satisfying the required power needs of ESP32 configuration requiring a total daily energy production of 35.1 Wh/day. Therefore, as a matter of design policy and operational reliability assurance, the floating sensing station incorporates an oversized monocrystalline solar array rated at 50 W. Under identical conditions as above, this solar array produces daily solar yield of 140 Wh/day, resulting in a daily solar surplus of 113.8 Wh/day. The battery bank will recover back to its full capacity within normal daylight cycles and prevent potential brownout conditions associated with long-term cloudy weather patterns. Additionally, the excess generated energy will directly offset high-current actuation demands for Case B (robotic support hub). High-current DC power is sent down a lengthy (∼50 m) tether to drive electrical-mechanical devices associated with mobility-based underwater robots, causing excessive battery drain. The large size of the solar array enables recovery of primary power reserves after extensive robotic support operations occur.

**Fig. 16:**
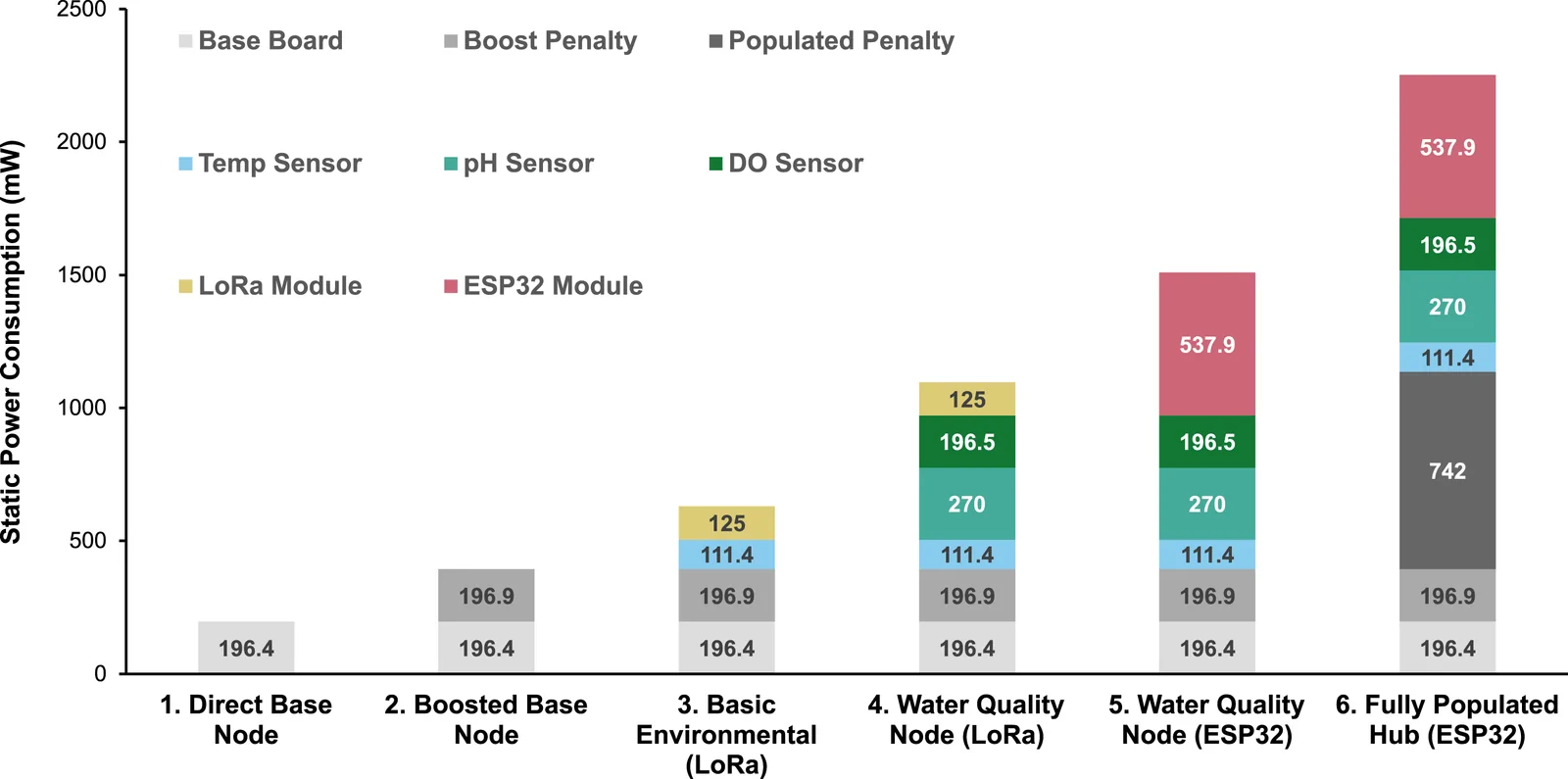
**Static Power Consumption Breakdown.** Stacked visualization of the isolated component-level milliwatt (mW) penalties across six discrete hardware configurations.

**Fig. 17:**
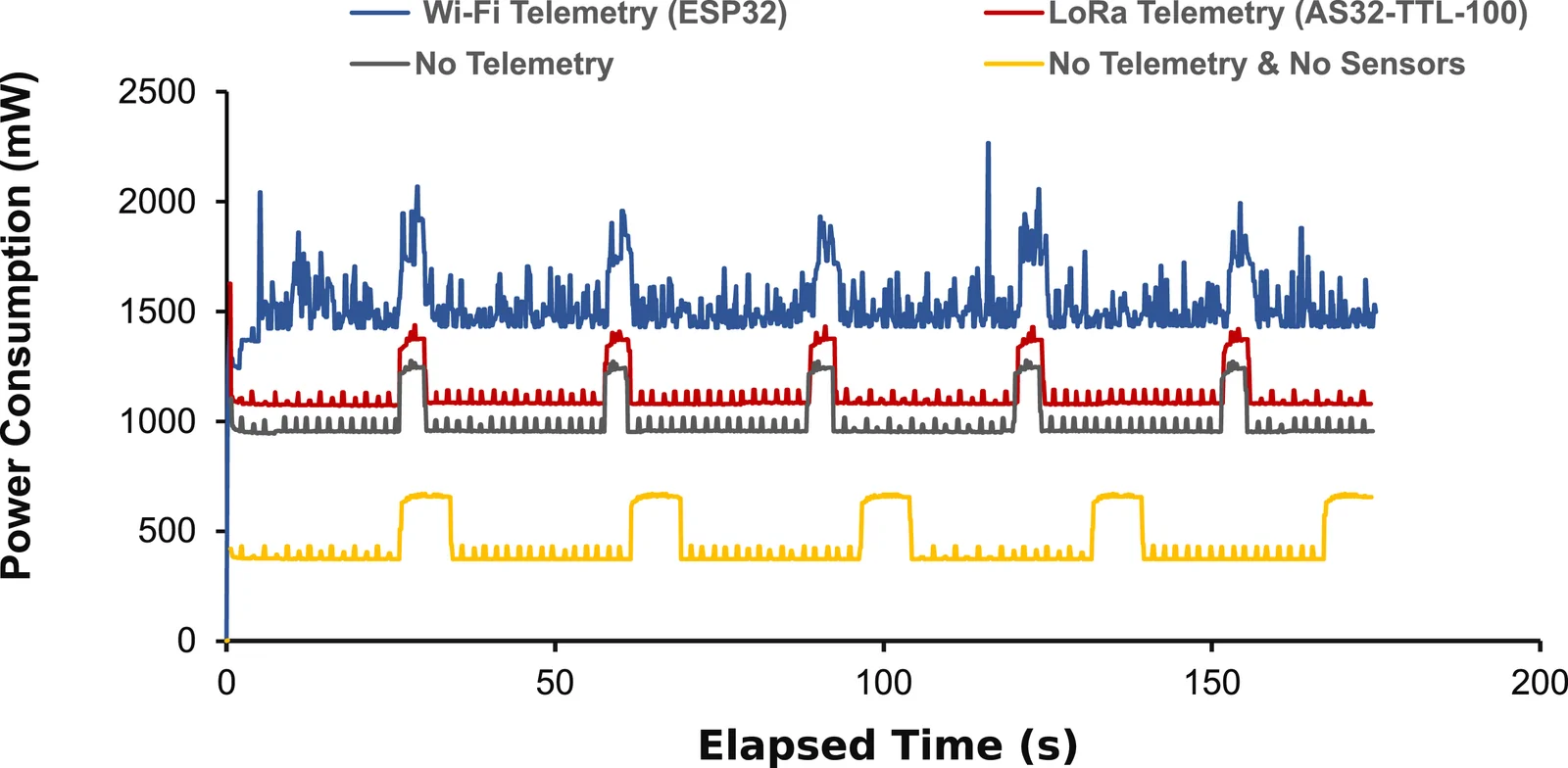
**Dynamic Power Profile.** High-resolution continuous power tracking across four hardware states. All plotted configurations use a semi-populated RS485 node connected to a boost converter (12 V input stepped up to 22 V output).

**Fig. 18:**
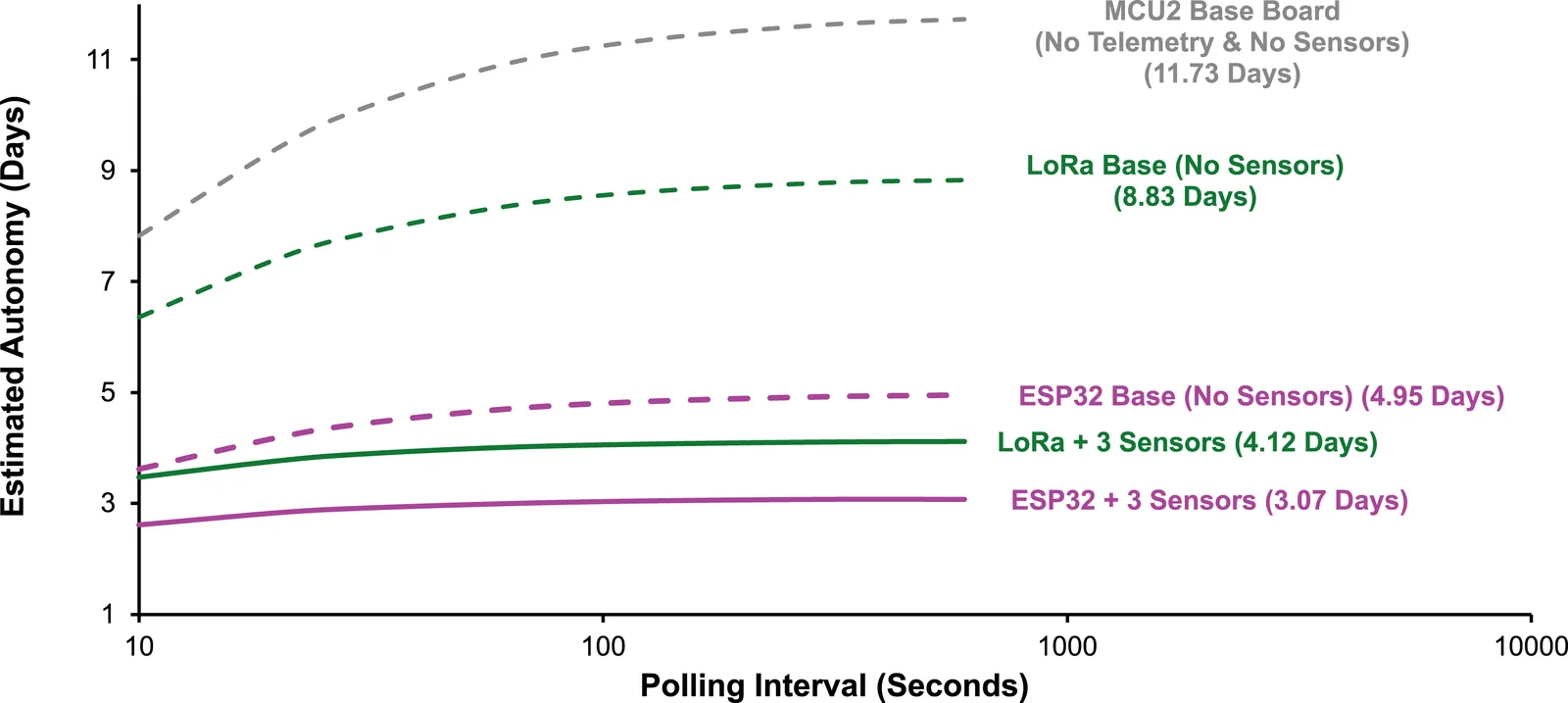
**Estimated System Autonomy with a 108 Wh Battery.** Projections assume a 7-second active duty cycle per 600-second polling interval. The plotted data represents continuous-power operation without sensor gating. **Note:** Implementing MOSFET high-side power gating neutralizes sleep-phase parasitic drain, projecting the multi-sensor configuration curves to approximate their respective sensorless baselines (LoRa Base and ESP32 Base).

### 7.2 Long-term endurance and stability

A ten-day continuous benchmarking test from May 25 through June 4 subjected the equipment to cyclic ambient temperature variation. This experiment was designed to evaluate the long term integrity of the RS-485 data bus as well as to monitor the stability of the telemetry system. The experimental method used strictly physical separation. The JXCT sensor arrays and a commercial reference meter (Renile) were placed into an undisturbed 9 L container filled with stagnant tap water. No mechanical agitation, artificial aeration or chemical treatment occurred during this ten day time period, maintaining a completely static environment.

The main variable factors that caused changes in the water’s physical states were the daily room temperature fluctuations due to the cycle on/off of an AC unit rated for 20 °C. These temperature swings created a thermal lag condition, where the water’s temperature was changing significantly faster than the rate of passive equilibrium that occurred as the trapped dissolved carbon dioxide equilibrated with the surrounding air. The OpenWater Hub continuously polled and transmitted Modbus RTU telemetry data every 34.1 s. On the other hand, the commercial reference device recorded its data internally at fixed one-hour intervals.

The analysis of the collected time-series data verifies that the system is in an operational state for long periods (see Fig. 19). Water was initially introduced into the system at a temperature of 10.5 °C. Over the next 24 h (while the AC was turned off) the stagnant water warmed through absorption of ambient heat to an equilibrium temperature of 24 °C. The OWH tracking of the thermodynamics indicated that it successfully followed this process. The initial non-linear increase in pH recorded by the OWH indicates that the physical out-gassing of dissolved CO_2_, which was contained in the water provided by the pressurized tap-water supply, occurred as the water reached chemical equilibrium with its atmospheric surroundings.

Environmental testing of the equipment continued over subsequent days. The prolonged activation of AC caused the water temperature to drop into a stable low band, with brief thermal spikes resulting from temporary airflow. A significant turning point in the test occurred on June 1st, when the AC was turned off, which caused an extended thermal increase until June 3rd, where it reached approximately 26.0 °C prior to the AC being turned on again.

Throughout the changes in the thermodynamics as described above, the OWH had an excellent level of telemetry data integrity. Of 25,314 packet transmissions generated by the hardware during the 240 h studied, 25,308 packets were recorded to the cloud-based database. The greater than 99.97% packet delivery ratio demonstrates that the ATmega128A dual-MCU Modbus design is stable. The six lost packets are attributed to temporary assignment of IP addresses and/or wireless route collisions with the ESP32 module; this loss does not indicate a single hardware halt of the RS485 polling bus at any time, nor did it indicate any hardware-related timeout communications over a period of ten days.

The tracking of the baseline for the still water body allowed us to develop an instrumentation drift map with the factory calibration setting for both sensors. Both the continuous OWH telemetry and the discrete data points from the commercial reference superimposed well upon one another. Additionally, the high-resolution (34.1 s) sampling of the OWH exposed transitory changes in the environment which were obscured due to the 1 h data export limit of the commercial reference.

**Fig. 19:**
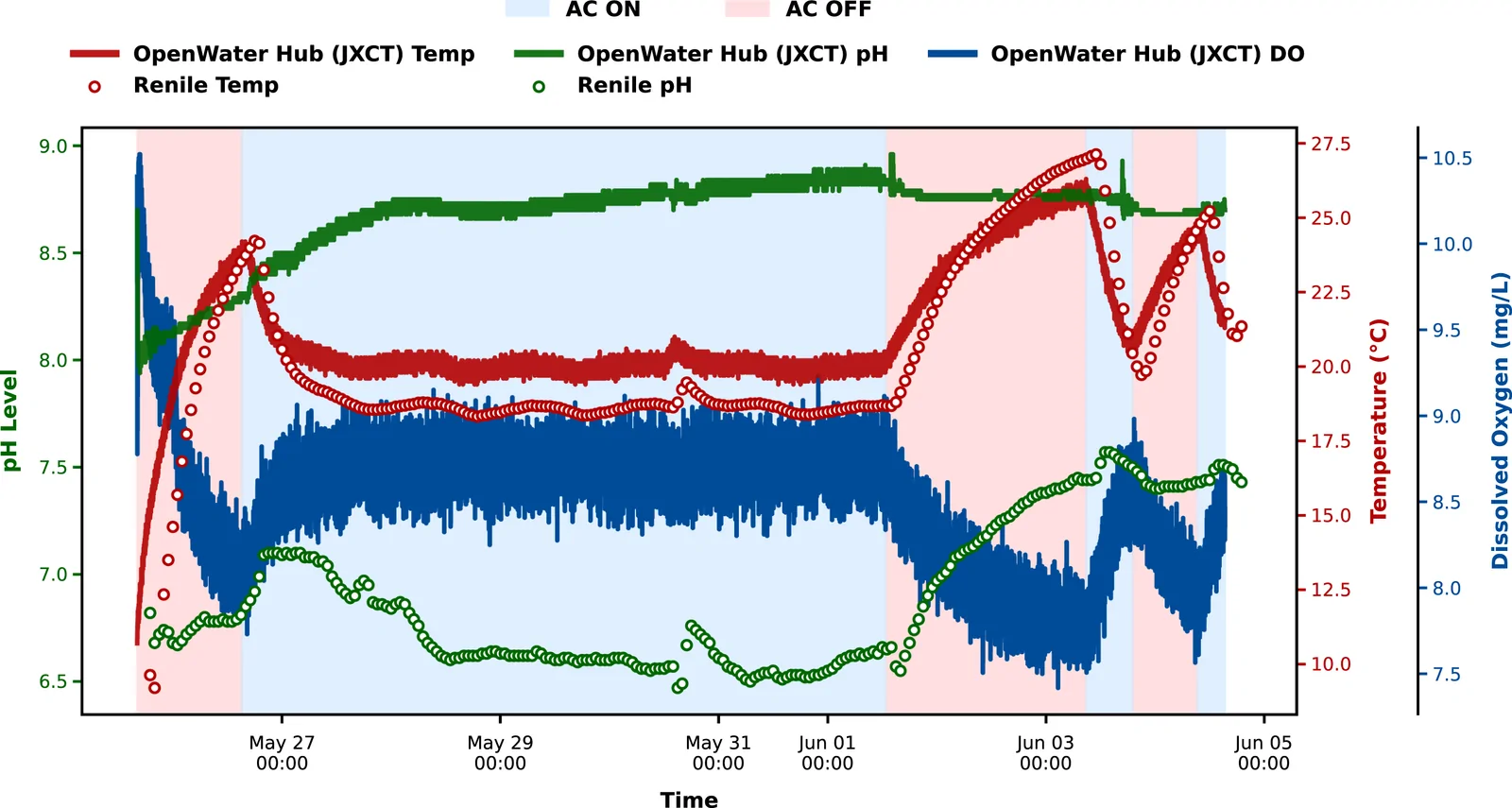
**240-Hour Endurance and Stability.** Time-series plot tracking pH, Temperature, and Dissolved Oxygen under cyclic ambient thermal loading. The continuous OpenWater Hub telemetry (34.1 s resolution (Adjustable)) is plotted alongside discrete data points from the commercial Renile reference (1 hr resolution (Non-Adjustable)).

### 7.3 Automatic temperature compensation analysis

The evaluation of the data logger involves the separation of the hardware’s ability to digitally capture signals from the physically based electrochemical process associated with each attached sensor. While measurement accuracy and operational limitations were completely dependent on the industrial sensors themselves, they did not limit in any way how well the communication hardware operated. The OpenWater Hub simply acted as an independent processor operating as a multi-node manager that executed RS485 communications and data logging independently of the attached probe’s chemical condition. The correlation analysis performed using the data captured over the course of the 240 h endurance test resulted in a graphical correlation plot showing temperature plotted against pH for the OWH-connected JXCT sensors and the Renile system (Fig. 20).

The pH in a thermodynamic sense drops slightly when you raise the temperature [74], [75]. The first part of the endurance test was conducted while the water warmed up to ambient room temperature, and at the same time it lost dissolved CO_2_ through physical outgassing. This removal of carbonic acid caused a rise in the basic pH for that environment and thus masked the normal drop in pH from increased temperature [76]. Each of these two sensor arrays recorded the temporary shift in the baseline.

Despite the fact that there are several chemical variables occurring at the same time in the data from OWH, the distribution of those data is randomly dispersed and has an R-squared ($R^{2}$) value of 0.0054. This negligible correlation between the two variables mathematically proves that the pH values acquired from the OpenWater Hub do not correlate to changes or trends in the cyclic temperature variations; and thus confirms that the automatic temperature compensation built into the JXCT industrial probe used for these measurements functioned properly during the slow thermal oscillations, and that the OWH accurately measured and transmitted those temperature-corrected measurements via RS485 communications without causing any digital artifacts.

The Renile reference dataset shows a significant, positive linear relationship (y=0.0807x+5.2399; $R^{2}=0.5591$) with the data points. The commercial trend line shows a pH value increase of approximately 0.08 units/°C, which is much larger than would be expected by thermodynamic variation of pH due to temperature changes. Therefore, it can be concluded that there was a thermal drift in that particular commercial probe over the duration of the test. This likely has more to do with the probe’s age, membrane damage, etc., vs. being a permanent hardware problem; another calibrated unit may also show no such baseline offset. This type of analysis isolates sensor calibration as an independent electrochemical variable so it confirms that the open-source electronics can acquire high-fidelity data from the external RS485 sensors regardless of what shape the probe is in.

**Fig. 20:**
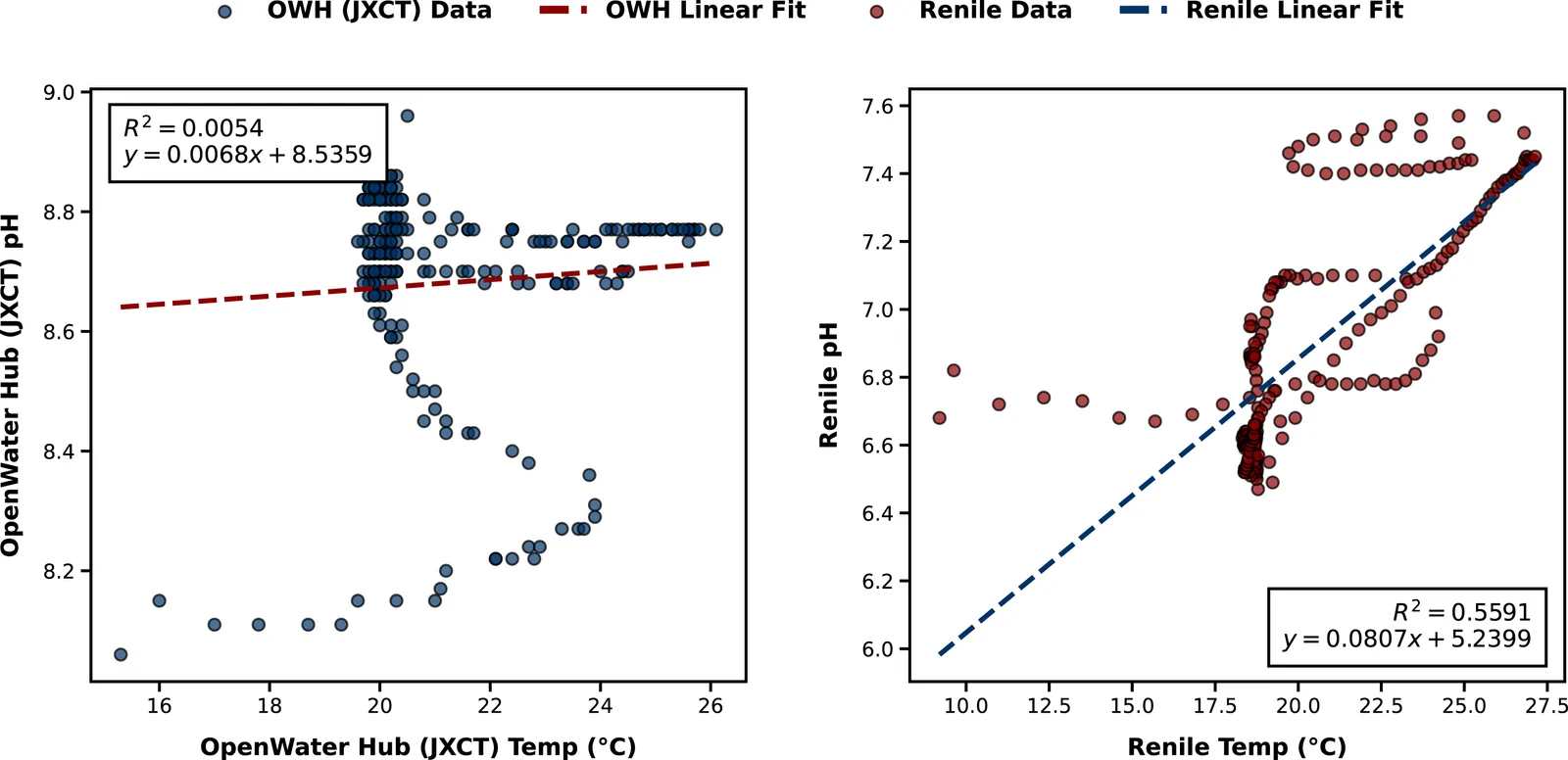
**Automatic Temperature Compensation Correlation.** Scatter plots defining the correlation between pH and temperature over the 10-day stability test. The left panel plots the JXCT sensors polled by the OpenWater Hub ($R^{2}=0.0054$). The right panel plots the commercial Renile reference sensors ($R^{2}=0.5591$).

### 7.4 Transient kinetics and system response

A 3.5-hour sequence subjected the RS485 bus to rapid chemical and mechanical perturbations. High-resolution logging captured transient responses across the full dynamic range of the sensors. This protocol quantifies MCU parsing latency against the commercial baseline through a series of isolated kinetic tests. Evaluation of mechanical degassing kinetics follows the activation of an 1800L/h pump (Fig. 21). Both architectures detect the pH elevation driven by physical CO_2_ stripping.

Sequential dosing with acetic acid and sodium bicarbonate maps the system’s chemical response curve (Fig. 22). The OWH resolves the acute pH drop and subsequent stepped recoveries. The acquired data matches the temporal response curve of the reference meter.

Temporal alignment verifies digital synchronization (Fig. 23). The protocol introduced heated saline solutions at specific intervals. The commercial reference logged an electrical conductivity (EC) spike. Simultaneously, the OWH logged a thermal spike.

Analysis of the transient pH dataset yields a linear regression model (Fig. 24). The resulting scatter plot demonstrates a correlation coefficient of r=0.9903 ($R^{2}=0.9807$). While Section 7.3 identified thermal drift over multi-day spans, this high correlation proves both systems track acute chemical shifts identically under stable thermal conditions. This metric mathematically validates the open-source telemetry layer. The hardware executes data transmission with high resolution. Baseline offsets remain strictly electrochemical variables.

**Fig. 21:**
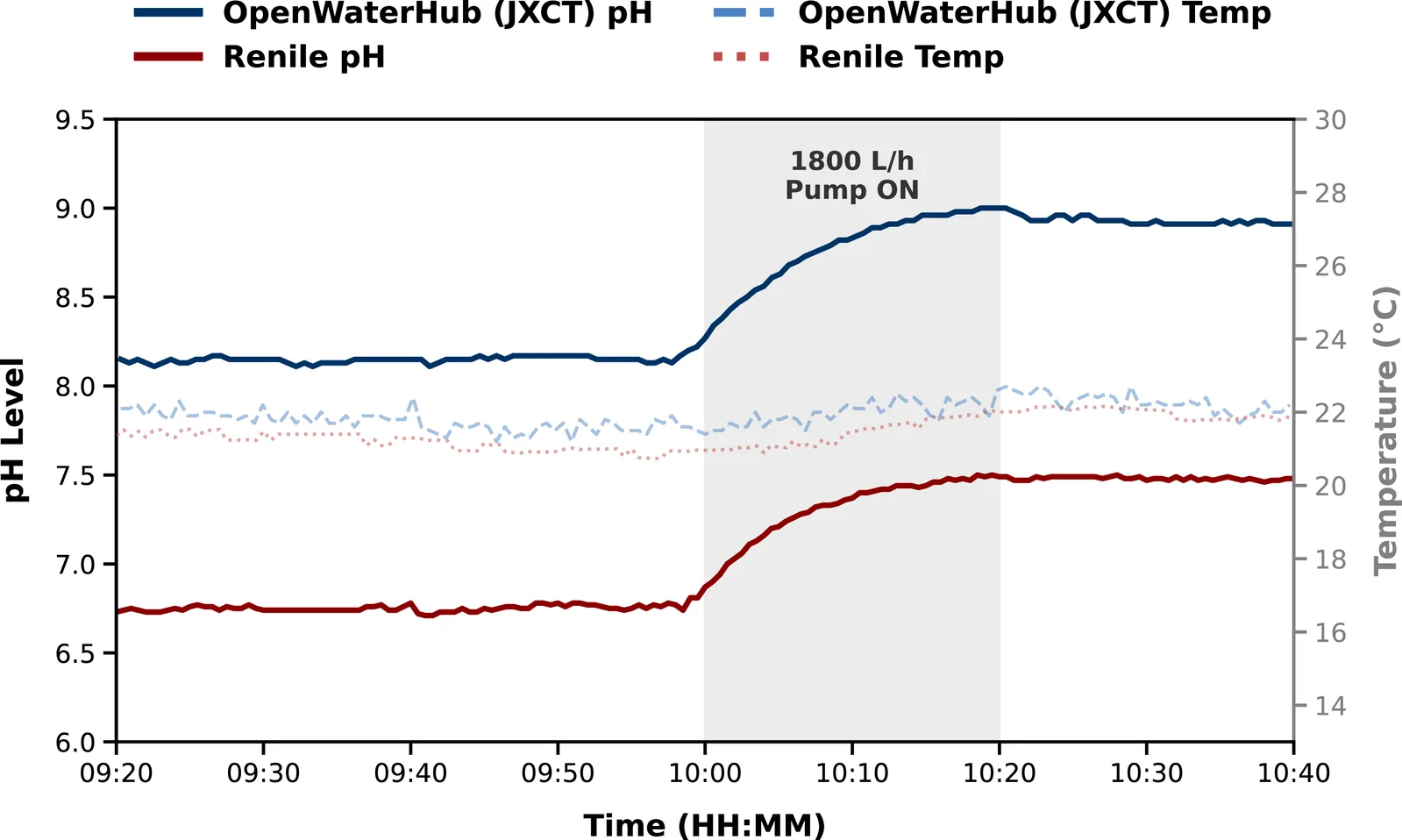
**Mechanical Degassing Response.** pH levels following mechanical CO_2_ degassing via an 1800L/h pump. Both systems correctly identify the physical gas exchange.

**Fig. 22:**
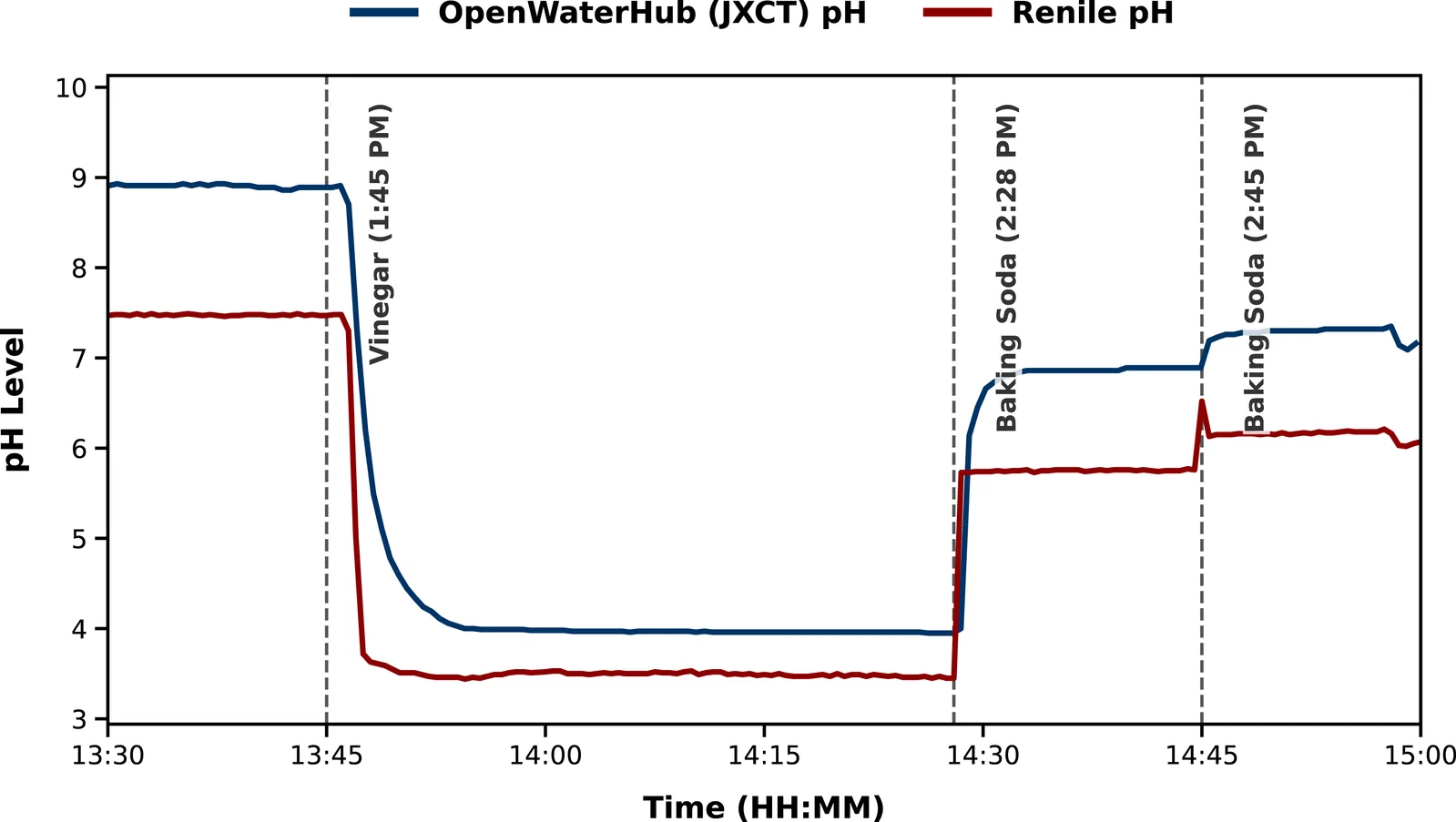
**Chemical Perturbation Tracking.** pH response to sequential acidic (acetic acid) and alkaline (sodium bicarbonate) perturbations, demonstrating the system’s dynamic range. OpenWater Hub Resolution: 34.1 s (Adjustable) vs Renile Resolution: 30 s (Non-Adjustable).

**Fig. 23:**
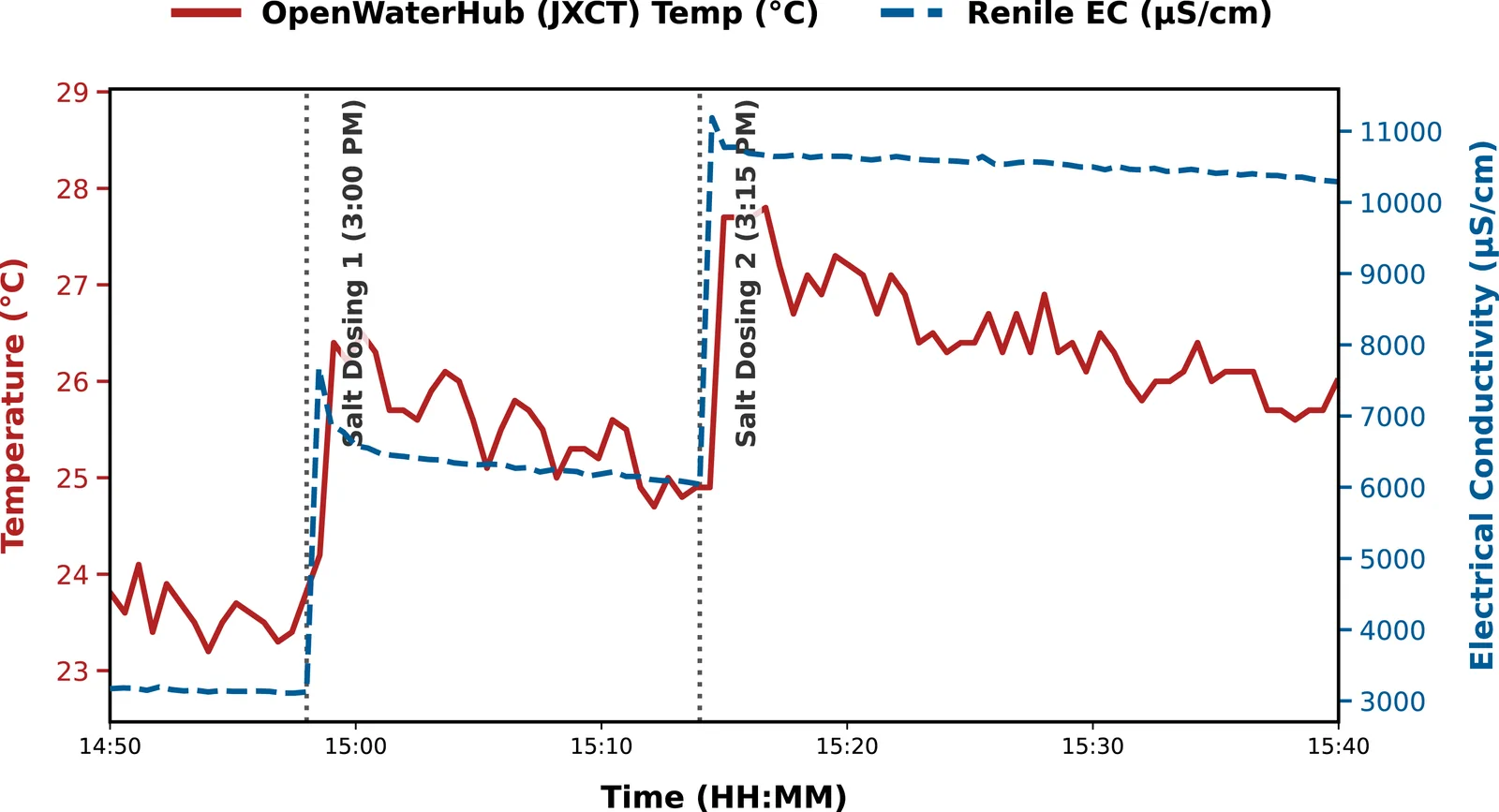
**Temporal Alignment.** Synchronization of heated saline dosing, plotting OpenWater Hub temperature against commercial reference electrical conductivity to verify zero-latency digital parsing.

**Fig. 24:**
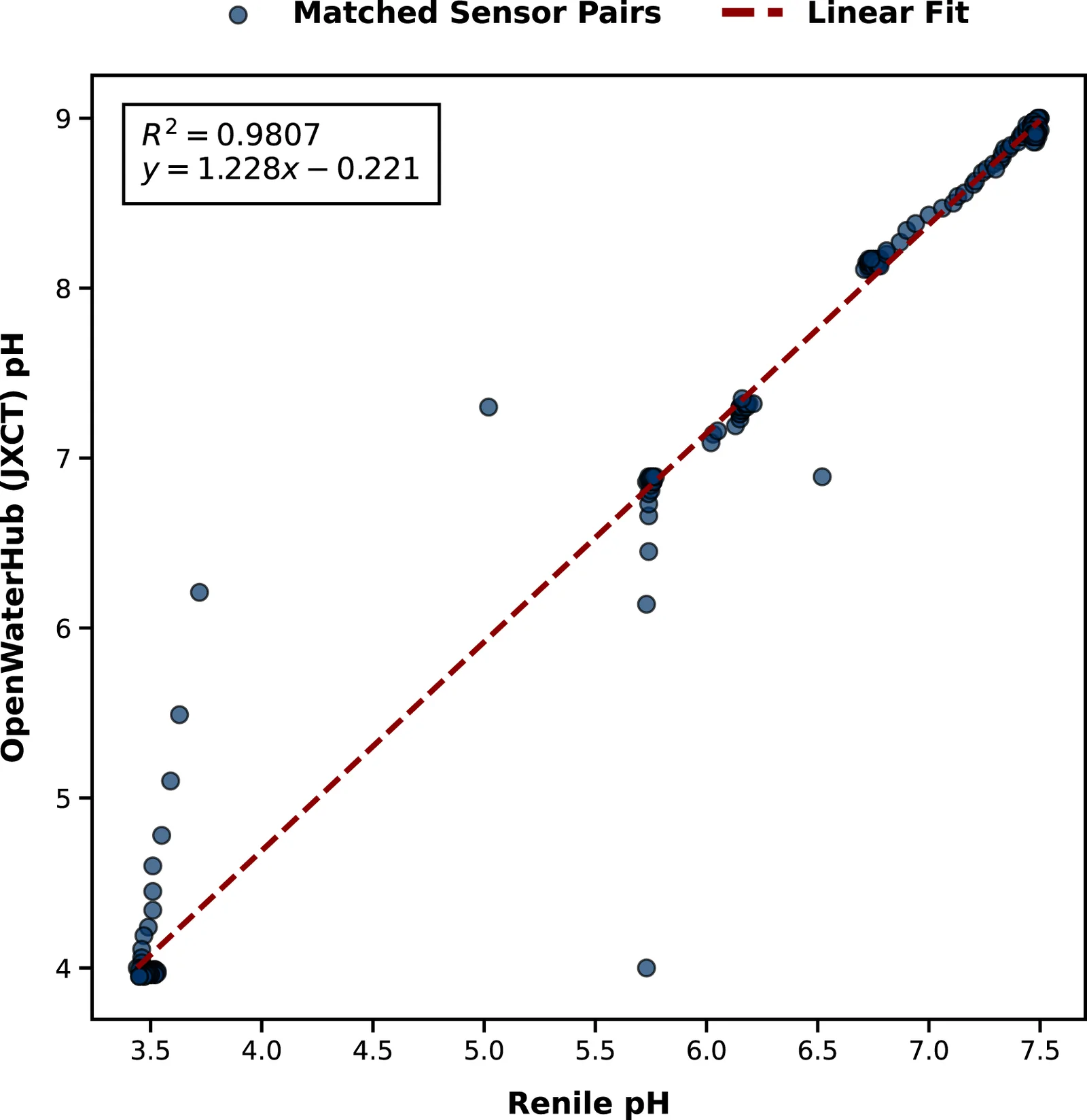
**Transient pH Correlation.** Linear regression model ($R^{2}=0.9807,r=0.9903$) of the high-resolution transient pH dataset, mathematically validating the digital parsing integrity of the open-source hardware.

### 7.5 System integration and robotic actuation

An 8 node RS-485 sensor array was tested to determine the full bus bandwidth of this system. The system polled each address sequentially in order from 1 through 8 with no timeouts or buffer overflows (see Fig. 27). Demonstrations of field portability and power independence were performed on pool side by utilizing the Case C configuration, transmitting parsed data sets from the sensors to a local Bluetooth terminal (see Fig. 25a). Physical integration and buoyancy testing of the Case A/B configurations were also conducted as part of a shallow coastal deployment (see Fig. 25d).

The long-range tethered telemetry system’s ability to send/receive commands via an RS-485 interface has been validated over a 50 m multicore cable. Power transmission will be removed from this specific bench test so as to allow isolation of the data bus. The hardware was able to transmit command packets over the 50 m and thus validate its ability to provide reliable support for robotics in open water (Fig. 25c).

A soft-actuated robotic fish was designed for laboratory testing as shown in Fig. 26. Tail actuators were driven by the boards PWM outputs. Onboard temperature and electrical conductivity sensors measured physical properties of water. Simultaneous real-time telemetry demonstrated no loss of RS485 communication between the robot’s onboard computer and its remote payload while continuously operating at high currents to operate the motors. Such a modular, multi-topology design allows the OpenWater Hub to support future modular robotic platforms. As an example, segmented, biomimetic robots (i.e., robotic fish) may have independent kinematic and buoyancy modules [77], which will be capable of interfacing with the OpenWater Hub using either of the two different topological configurations. For example, depending upon the desired level of automation and/or test environment requirements, the OpenWater Hub can relay commands to the onboard robot controllers via the RS485 communication link. Alternatively, when time is important during bench tests or controlled lab tests, the OpenWater Hub could bypass the robot controller altogether to directly drive each pectoral and tail actuator via high-current PWM output. However, since PWM signals are severely attenuated by longer lengths of cable, direct actuation is restricted to short-range applications only. Longer distance deployments would therefore need to utilize the command relay functionality provided by RS485.

**Fig. 25:**
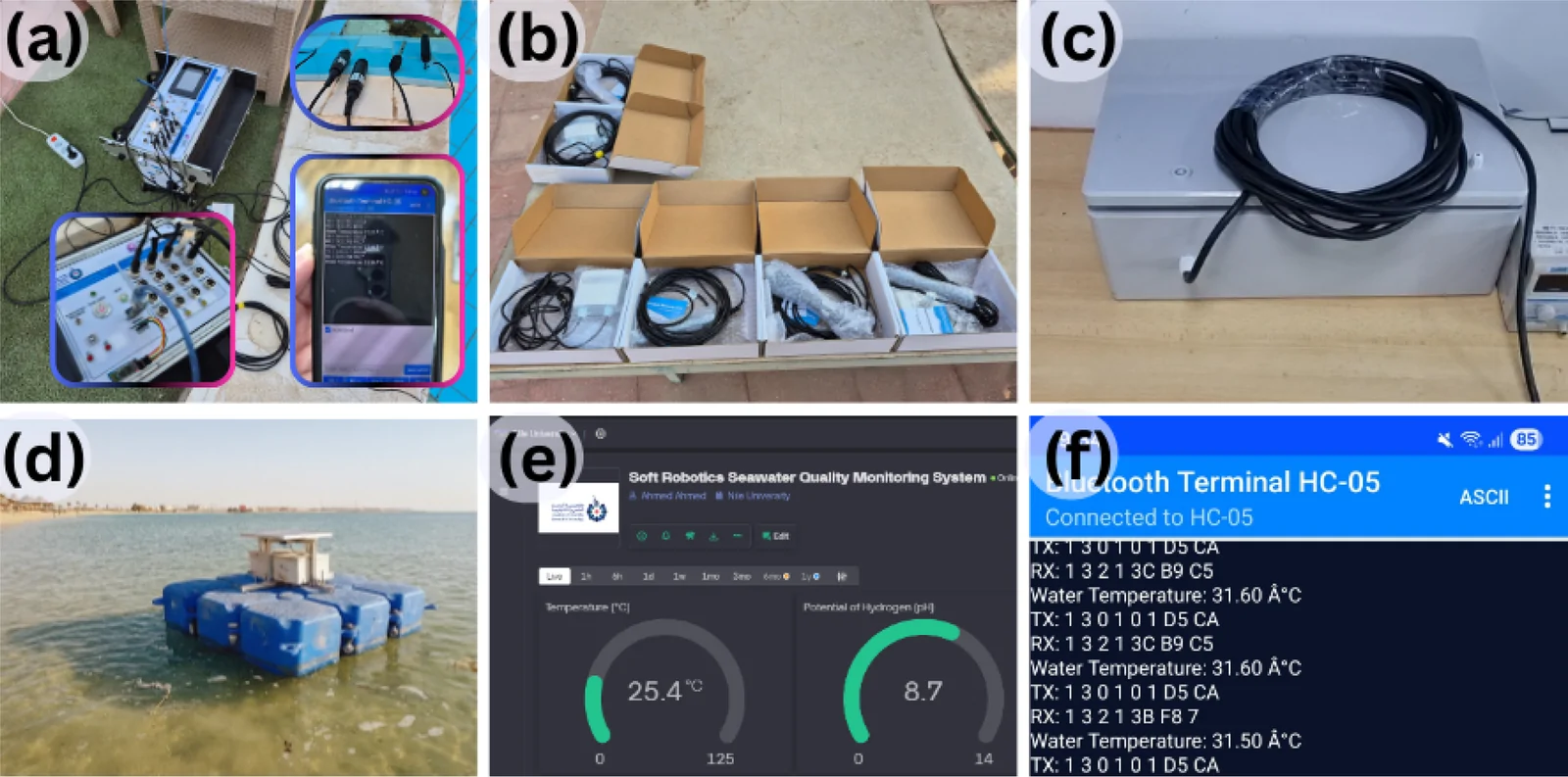
**Comprehensive System Validation.** (a) Portable configuration connected to four submerged JXCT probes, broadcasting via Bluetooth. (b) Six-node JXCT RS485 sensor array. (c) Floating Station IP66 enclosure featuring the 9-pin tether interface. (d) Shallow coastal deployment demonstrating intended physical integration and platform buoyancy. (e) Blynk IoT dashboard displaying transmitted telemetry. (f) Raw serial data output via the HC-05 module.

**Fig. 26:**
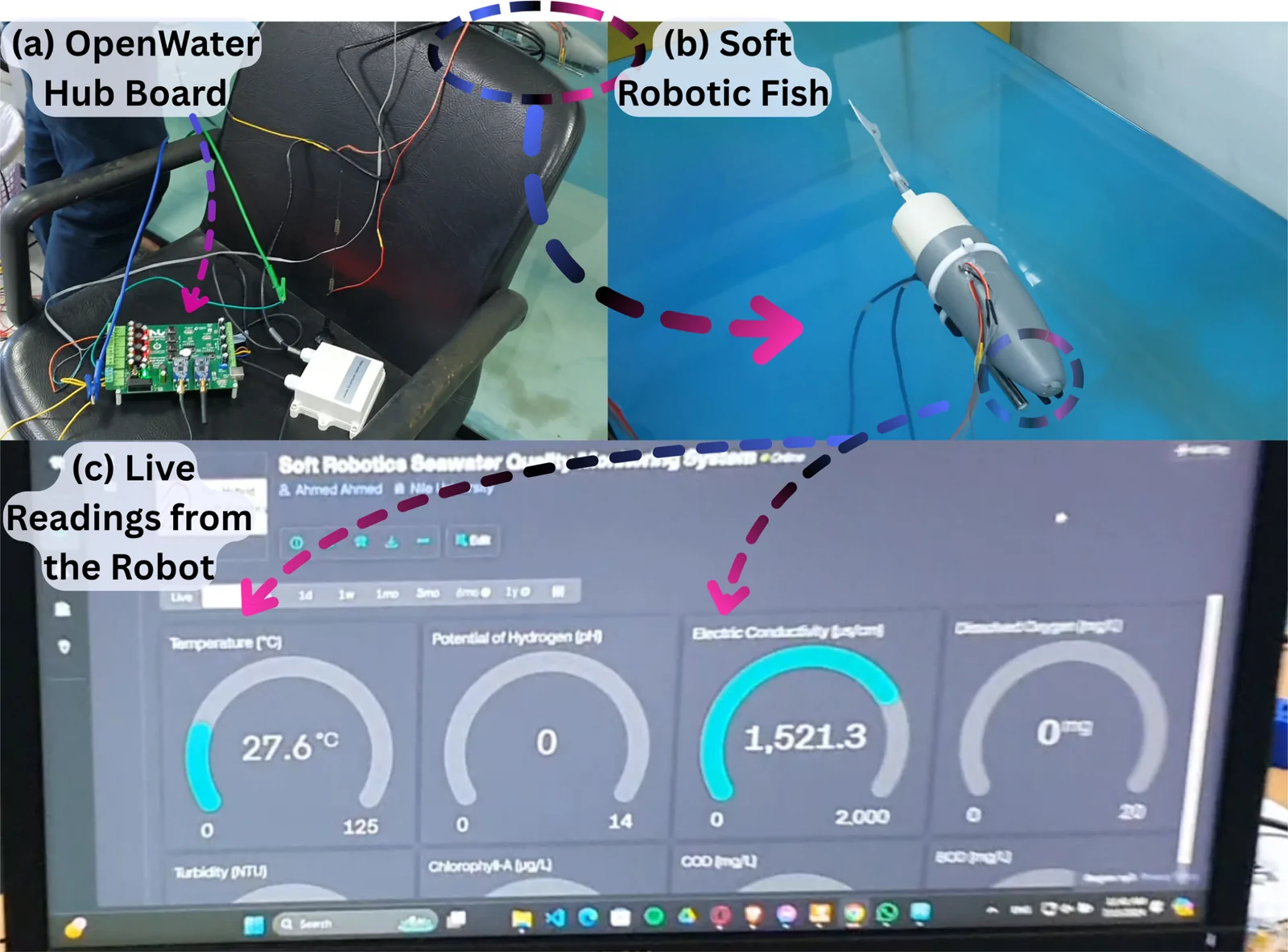
**Direct Control of a Soft Robotic Fish.** (a) OpenWater Hub tether interface. (b) Soft robotic fish deployed in a test tank, with tail actuators driven directly via PWM. (c) Dashboard tracking environmental data (temperature and EC) acquired from the mobile robot concurrently with motor actuation.

**Fig. 27:**
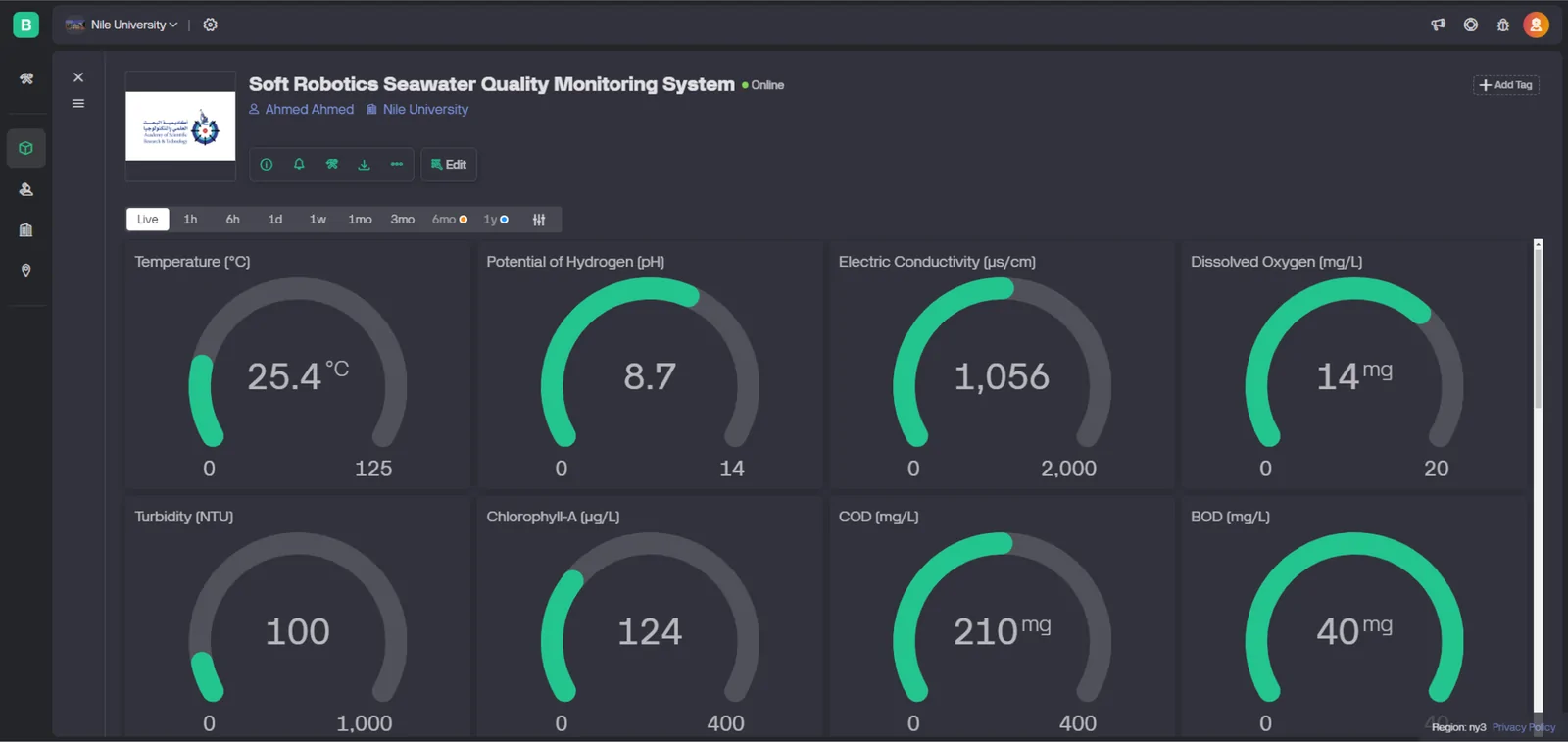
**Full-Spectrum Water Quality Monitoring.** Dashboard displaying aggregated real-time data from an 8-node RS485 sensor array (temperature, pH, EC, DO, turbidity, Chl-a, COD, and BOD).

### 7.6 Conclusions, limitations, and future work

#### 7.6.1 Conclusions

The OpenWater Hub takes industrial sensors from closed systems and creates a deterministic, multi-processor control node. By using two MCUs, it eliminates serial latency caused by a single core. MCU2 is used for all things related to RS485 Modbus polling, local CRC16 error checking, and transceiver timing within a dedicated bare-metal blocking loop. In the meantime, MCU1 will run time-critical kinematics. A fused 1.0 A, 24 V direct pass-through bus supplies up to 24 W of on-board power to supply the high inrush currents demanded by the mechanical wiper motors of optical sondes. Isolated from this sensor bus are four independent step-down regulators that allow for PWM actuation channels. They have an adjustable 8.85 V output and can be set to support up to 3.0 A. The open-loop configuration allows the MCU1 pin to control brushless DC electronic speed controllers (ESCs). Also, the operator can connect these pins to external MOSFETs, allowing for the control of typical DC motors, heavy water pumps, and hydroponics/aquaculture flow valves.

#### 7.6.2 Limitations

This topological isolation introduces unique engineering constraints. Although some modern RS485 sensing nodes have the capability to receive software sleep command requests, achieving truly zero quiescent power usage is dependent on physical control. An operator must therefore insert an additional high-side P-channel MOSFET into the sensor wiring (see Section 2.1.4) for true zero quiescent power control. The onboard regulators (LM2576) will strictly limit silicon current output to 3.0 amps/channel. In general, this level of silicon current limitation will be suitable for short tethered soft robot actuators, or for typical flow valve applications; however, if you are planning to drive heavy surface mechatronic devices such as automated sensor winch systems, or high capacity sampling pump systems, you may need to bypass these internal regulators, and connect your high current converter(s) directly to the main bus. Architecturally, the shared ground plane limits the system’s ability to provide galvanic optical isolation. If water enters through either the connectors or cable insulation, and in conductive seawater abrades away, it can create ground loops that skew sensitive electrochemical measurements. Additionally, due to the legacy nature of the 8-bit processing architecture, complex onboard digital signal processing cannot be performed by the MCU since it does not contain the necessary hardware floating-point units to perform wave spectrum calculations. Finally, although physically separating the copper data traces for MCU1 and MCU2 prevents motor electrical interference with serial telemetry, this separation also forces operators to select either a wireless LoRa bridge option or to add a physical UART jumper, which is dependent upon operators having basic programming and electronics knowledge.

#### 7.6.3 Future work

Future developments in this field should be developed with a shift from controlled aquatic test sites to open sea. Long term exposure to marine environments is required for assessing the durability of marine systems; therefore, continued operation of the sensor system under these conditions will provide data on the structural degradation caused by continuous wave impact and saltwater corrosion on both the aluminum truss frame supporting the sensors and the IP67 cable glands. Additionally, the need to empirically demonstrate the ability to maintain stability over long periods of time necessitates further experimentation regarding the effects of biofouling on the mechanical integrity of the structure. In addition to demonstrating that the structure can remain physically effective throughout a multi-month deployment window, subsequent experiments will determine whether commercially available (third-party) mechanical wiping devices are sufficient to remove the biological material from the surface of the sensors and, if so, how frequently such cleaning would be required. Software support continues to represent an important part of ongoing operational planning. The authors plan to continue updating the open-source code repository where users may find updates to resolve issues with firmware or report updated state machine routines related to the integration of additional industry payloads within the overall system.

## CRediT authorship contribution statement

**Hany A. Elesawy:** Writing – review & editing, Writing – original draft, Visualization, Validation, Software, Methodology, Investigation, Data curation, Conceptualization. **Ahmed I. Ahmed:** Writing – review & editing, Writing – original draft, Visualization, Validation, Software, Methodology, Conceptualization. **Yahia A. AboZaid:** Writing – review & editing, Writing – original draft, Visualization, Validation, Methodology, Investigation, Conceptualization. **Lobna A. Said:** Writing – review & editing, Supervision, Project administration. **Irene Samy:** Writing – review & editing, Supervision, Resources, Project administration, Conceptualization. **Ahmed G. Radwan:** Writing – review & editing, Supervision, Resources, Project administration, Funding acquisition, Conceptualization.

## Ethics statements

This research did not involve human subjects, animal experiments, or any other ethical concerns requiring specific approval.

## Declaration of competing interest

The authors declare that they have no known competing financial interests or personal relationships that could have appeared to influence the work reported in this paper.

## References

[1] Duarte C.M., Agusti S., Barbier E., Britten G.L., Castilla J.C., Gattuso J.-P., Fulweiler R.W., Hughes T.P., Knowlton N., Lovelock C.E., Lotze H.K., Predragovic M., Poloczanska E., Roberts C., Worm B. (2020). Rebuilding marine life. Nature.

[2] Moberg F., Folke C. (1999). Ecological goods and services of coral reef ecosystems. Ecol. Econom..

[3] Gaines S., Cabral R., Free C.M., Golbuu Y., Arnason R., Battista W., Bradley D., Cheung W., Fabricius K., Hoegh-Guldberg O., Juinio-Meñez M.A., Molinos J.G., Ojea E., O’Reilly E., Turley C., Lubchenco J., Haugan P.M. (2023). The Blue Compendium.

[4] Silva G.M.E., Campos D.F., Brasil J.A.T., Tremblay M., Mendiondo E.M., Ghiglieno F. (2022). Advances in technological research for online and in situ water quality monitoring—A review. Sustainability.

[5] Batur E., Maktav D. (2019). Assessment of surface water quality by using satellite images fusion based on PCA Method in the Lake Gala, Turkey. IEEE Trans. Geosci. Remote Sens..

[6] Koparan C., Koc A., Privette C., Sawyer C. (2018). In situ water quality measurements using an Unmanned Aerial Vehicle (UAV) System. Water.

[7] Melo M., Mota F., Albuquerque V., Alexandria A. (2019). Development of a robotic airboat for online water quality monitoring in lakes. Robotics.

[8] Demetriou G., Lazaridis S., Trimithiotis G., Christofi M., Louka A., Alkiviadous S. (2025). Open-Source BlueOS for Controlling the SeaBot USV. IOP Conf. Ser.: Earth Environ. Sci..

[9] Wu Z., Liu J., Yu J., Fang H. (2017). Development of a novel robotic dolphin and its application to water quality monitoring. IEEE/ASME Trans. Mechatronics.

[10] Liu J., Zhao J., Liu Z., Liu Y., Ren Y., An D., Wei Y. (2025). Development and application of the coverage path planning based on a biomimetic robotic fish. J. Field Robot..

[11] Katzschmann R.K., DelPreto J., MacCurdy R., Rus D. (2018). Exploration of underwater life with an acoustically controlled soft robotic fish. Sci. Robot..

[12] Van Den Berg S.C., Scharff R.B., Rusák Z., Wu J. (2022). OpenFish: Biomimetic design of a soft robotic fish for high speed locomotion. HardwareX.

[13] Ligman M., Rezaie K.A., Shah R., Keeter C., Sutterfield B., Fürth M. (2025). Fluid interaction study: Hydrodynamic robot (FISHR) — Expansion of bioinspired soft robotic fish. HardwareX.

[14] Toma D.M., del Rio J., Jirka S., Delory E., Pearlman J. (2014). 2014 IEEE Sensor Systems for a Changing Ocean (SSCO)..

[15] Soltero C. (2017).

[16] Kim H.J., Lee M.H. (2026). A Comparative Performance Study of Parallel MQTT and RS485 Communication Architectures for High-Frequency IoT Sensing. Electronics.

[17] Sun C., Guo K., Xu Z., Ma J., Hu D. (2019). 2019 Chinese Automation Congress (CAC).

[18] Mancheno D., Gamboa S., Botto-Tobar M., Zambrano Vizuete M., Diaz Cadena A., Vizuete A.Z. (2022). Latest Advances in Electrical Engineering, and Electronics.

[19] Chen Y.-J., Hsu H.-W. (2022). 2022 14th International Conference on Computer and Automation Engineering (ICCAE).

[20] Vincent P.S., McMahon P.J., Muscat R.F., Zeve L., Wilson A.R. (2007). Smart Structures, Devices, and Systems III.

[21] M.H. Schraven, C. Guarnieri Calo Carducci, M.A. Baranski, D. Müller, A. Monti, Designing a development board for research on IoT applications in building automation systems, in: 36th International Symposium on Automation and Robotics in Construction, Banff, AB, Canada, 2019, 10.22260/ISARC2019/0012.

[22] Cuomo M. (2022). OCEANS 2022, Hampton Roads.

[23] Agarwal A., Cohn D. (2020). Global Oceans 2020: Singapore – U.S. Gulf Coast.

[24] Peng J., Hao R., Zhang L., Pan C., Yu J., Gao X. (2022). 2022 3rd International Conference on Electronics, Communications and Information Technology (CECIT).

[25] DaSilva S.J., Cleland J., Chappell S.G., Graves M. (2009). OCEANS 2009.

[26] Turner M.W., Cleland J.G., Baker J. (2009). OCEANS 2009.

[27] Ahsan L., Baig M.J.A., Iqbal M.T. (2022). Low-Cost, Open-Source, Emoncms-Based SCADA System for a Large Grid-Connected PV System. Sensors.

[28] Mohamed Hariri M.H., Din A.S., Arshad M.R., Mat Desa M.K., Abdullah M.N. (2023). 2023 IEEE IAS Global Conference on Renewable Energy and Hydrogen Technologies (GlobConHT).

[29] YSI EXO3 Multiparameter Sonde, https://www.ysi.com/exo3?srsltid=AfmBOorXiOKe1NIvRrEbDVQMHspsltBrQ2hePYZNkIW-lac5bvjytTQq.

[30] Y4002 Integrated Multi-parameter Sonde-Water Quality Sensor Manufacturer-Yosemitech, https://www.yosemitesensors.com/multiparameter/Y4002-Integrated-Multi-parameter-Sonde.html.

[31] CB-75 Data Buoy, https://www.nexsens.com/products/data-buoys/cb-75-data-buoy.

[32] X3 Environmental Data Logger, https://www.nexsens.com/products/data-loggers/x3-environmental-data-logger.

[33] Aqualabo LowTuS, https://www.aqualabo.fr/en/produit/lowtus-2/.

[34] VuLink remote telemetry monitoring | Continuous Water Monitoring | In-Situ, https://in-situ.com/en/vulink/.

[35] SenseCAP S2100 LoRaWAN Data Logger | Seeed Studio Wiki, https://wiki.seeedstudio.com/Sensor/SenseCAP/SenseCAP_Data_Logger/Data_Logger/.

[36] Hawk Pro Data Logger – Flexible IoT Data Logger for Remote Monitoring, https://sense.digitalmatter.com/hawk-pro-data-logger.

[37] Water Quality Sensors, Meters, and Measurement Systems, https://www.ysi.com/products?srsltid=AfmBOopQjaKnZ8DG1yGz22HvDGgv7jS2mhCtwnn46h6yD5Fe771Mr1Nj.

[38] Mouy X., Black M., Cox K., Qualley J., Mireault C., Dosso S., Juanes F. (2020). FishCam: A low-cost open source autonomous camera for aquatic research. HardwareX.

[39] Zahari N.M., Sankar P., Zawawi M.H., Roslin N.T., Aziz N.A., Nurhikmah F., Khamarudin M.H.F., Sabtu N. (2024). Proceedings of AWAM International Conference on Civil Engineering 2022—Volume 2.

[40] Rabault J., Nose T., Hope G., Müller M., Breivik Ø., Voermans J., Hole L., Bohlinger P., Waseda T., Kodaira T., Katsuno T., Johnson M., Sutherland G., Johansson M., Christensen K., Garbo A., Jensen A., Gundersen O., Marchenko A., Babanin A. (2022). OpenMetBuoy-v2021: An easy-to-build, affordable, customizable, open-source instrument for oceanographic measurements of drift and waves in sea ice and the open ocean. Geosciences.

[41] Wickert A.D., Sandell C.T., Schulz B., Ng G.-H.C. (2019). Open-source Arduino-compatible data loggers designed for field research. Hydrol. Earth Syst. Sci..

[42] Agade P., Bean E. (2023). GatorByte – An Internet of Things-Based Low-Cost, Compact, and Real-Time Water Resource Monitoring Buoy. HardwareX.

[43] Richards W., Selker J., Udell C. (2024). Loom: A modular open-source approach to rapidly produce sensor, actuator, datalogger systems. Sensors.

[44] hydromisc (2026). https://github.com/hydromisc/hydromisc.

[45] Thomson J., Bush P., Castillo Contreras V., Clemett N., Davis J., De Klerk A., Iseley E., Rainville E.J., Salmi B., Talbert J. (2024). Development and testing of microSWIFT expendable wave buoys. Coast. Eng. J..

[46] Yurovsky Y.Y., Kudinov O.B. (2026). A very small wave buoy: The challenges learned from a proof-of-concept field experiment. Ocean Eng..

[47] Trevathan J., Schmidtke S. (2022). Open-source Internet of Things remote aquatic environmental sensing. HardwareX.

[48] Kodaira T., Katsuno T., Nose T., Itoh M., Rabault J., Hoppmann M., Kimizuka M., Waseda T. (2024). An affordable and customizable wave buoy for the study of wave-ice interactions: Design concept and results from field deployments. Coast. Eng. J..

[49] Hayibo K.S., Pearce J.M. (2025). Jericho open resistive data logger: An open-source modular weather station and monitoring system for long-term solar photovoltaic outdoor experimentation. HardwareX.

[50] Amorim M.L.M., Ando Junior O.H., Gazziro M., do Carmo J.P.P. (2026). Nexus: A modular open-source multichannel data logger—architecture and proof of concept. Automation.

[51] Nguyen B., Goto B., Selker J.S., Udell C. (2021). Hypnos board: A low-cost all-in-one solution for environment sensor power management, data storage, and task scheduling. HardwareX.

[52] Waldmann C., Meckel S., Huber R., Montenegro S., Ruffer M., Becker M. (2019). OCEANS 2019 - Marseille.

[53] Sládek I., Skovajsa M., Kuchár P., Kafková J., Šedivý Š., Gašpar G. (2025). A Universal I2C-to-RS-485 Module for Industrial Sensing. Electronics.

[54] Dafare M., Waghmare S., Bhoyar A., Titarmare A.S., Chandankhede P. (2023). 2023 International Conference on Circuit Power and Computing Technologies (ICCPCT).

[55] Cuenca-Sánchez A., Iza J., Proaño P., Valenzuela J. (2025). AI-Enhanced Embedded IoT System for real-time industrial sensor calibration. Eng. Proc..

[56] Jones J.P. (2024). 2024 IEEE Global Humanitarian Technology Conference (GHTC).

[57] Chrismettal (2026). https://github.com/Chrismettal/PiPLC.

[58] (2025). https://github.com/inoa-io/satellite.

[59] (2026). https://github.com/Atlantis-Specialist-Technologies/CAN485.

[60] Blanco-Jáquez A.D., Martínez-Cruz D.A., Alarcón-Herrera M.T., Marín-Celestino A.E., Valenzuela-García L.I. (2026). ESPiezometer: ESP32-based field tool for installation and validation of piezometric sensors for groundwater level monitoring. HardwareX.

[61] (2023). https://github.com/EnviroDIY/Mayfly-Modbus-Wing.

[62] Bristlemouth Dev Kit | Open Ocean Sensor Standard | Sofar Ocean, https://www.sofarocean.com/products/spotter/sensors/bristlemouth-development-kit.

[63] (2025). https://github.com/eChook/BMS-Comms-Board.

[64] PionixPublic/Reference-Hardware: BelayBox Reference Hardware, https://github.com/PionixPublic/reference-hardware.

[65] Complete Analysis - Portable Multiparameter, https://www.aqualabo.fr/en/sensors-and-measuring-equipment/measuring-devices/handheld-multiparameter/.

[66] Smart Mooring System Specs | Spotter with Smart Mooring | Sofar Ocean, https://www.sofarocean.com/products/spotter/smart-mooring/specs.

[67] Details and Specs – EnviroDIY, https://www.envirodiy.org/mayfly/hardware/details-and-specs/.

[68] ST-500 Inline PTSA Sensor, https://www.pyxis-lab.com/product/st-500-inline-ptsa-sensor/.

[69] Water Quality PH / ORP Measurement Electrode Liquid PH Sensor Controller-JXCT – Born for IoT, https://www.jxct-iot.com/product/showproduct.php?id=89.

[70] Water Monitoring for pH, DO, Flow and Quality | ReNile, https://renile.net/en/modules/water.

[71] Industrial-Grade MODBUS RS485 NH3 Sensor - with Waterproof Aviation Connector, https://www.seeedstudio.com/RS485-NH3-Sensor-Connector-p-5113.html.

[72] RS485 Industrial ORP Sensor, IP68, ModBus-RTU for Pro Water IoT, https://www.dfrobot.com/product-3102.html.

[73] Water Quality Sensor Manufacturer, Water Monitoring Sensors | Rika Sensor, https://www.rikasensor.com/water-quality-sensor.html.

[74] Govind Rajan A., Martirez J.M.P., Carter E.A. (2021). Coupled effects of temperature, pressure, and pH on water oxidation thermodynamics and kinetics. ACS Catal..

[75] Reineke K., Mathys A., Knorr D. (2011). Shift of pH-Value During Thermal Treatments in Buffer Solutions and Selected Foods. Int. J. Food Prop..

[76] Choi J., Hulseapple S., Conklin M., Harvey J. (1998). Modeling CO2 degassing and pH in a stream–aquifer system. J. Hydrol..

[77] Hamoud M., Elwahsh M.A., Sadek K.I., Shaaban N.K., Nooreldeen M.M., Donia A.N., Alsayed S.A., Mohamed O.A., Shaker A.K., Sayed H.A., Hamza A.M., Elesawy H.A., Fahim I.S., Radwan A.G. (2026). ZTUNA modular bionic fish design and manufacturing. Discov. Mech. Eng..

